# Passion Fruit Green Spot Virus Genome Harbors a New Orphan ORF and Highlights the Flexibility of the 5′-End of the RNA2 Segment Across Cileviruses

**DOI:** 10.3389/fmicb.2020.00206

**Published:** 2020-02-14

**Authors:** Pedro Luis Ramos-González, Gustavo Francisco dos Santos, Camila Chabi-Jesus, Ricardo Harakava, Elliot W. Kitajima, Juliana Freitas-Astúa

**Affiliations:** ^1^Instituto Biológico, Unidade Laboratorial de Referência em Biologia Molecular Aplicada, São Paulo, Brazil; ^2^PPG Microbiologia Agrícola Escola Superior de Agricultura Luiz de Queiroz, Universidade de São Paulo, Piracicaba, Brazil; ^3^Núcleo de Apoio à Pesquisa em Microscopia Eletrônica Aplicada a Agricultura, Escola Superior de Agricultura Luiz de Queiroz, Universidade de São Paulo, Piracicaba, Brazil; ^4^Embrapa Cassava and Fruits, Cruz das Almas, Brazil

**Keywords:** *Kitaviridae*, Negevirus-like lineage, Nelorpivirus, HTS, arthropod-infecting single-strand positive RNA viruses, *Passiflora* spp.

## Abstract

Passion fruit green spot and passion fruit sudden death are two reportedly distinct viral diseases that recurrently affect passion fruit (*Passiflora* spp.) groves in Brazil. Here we used a systematic approach that interconnects symptoms, transmission electron microscopy, RT-PCR detection assays followed by Sanger sequencing, and high-throughput sequencing of the RNA of affected passion fruit plants to gain insights about these diseases. Our data confirmed not only the involvement of cileviruses in these two pathologies, as previously suggested, but also that these viruses belong to the same tentative species: passion fruit green spot virus (PfGSV). Results revealed that PfGSV has a positive-sense RNA genome split into two molecules of approximately 9 kb (RNA1) and 5 kb (RNA2), which share about 50–70% nucleotide sequence identity with other viruses in the genus *Cilevirus*. Genome sequences of five PfGSV isolates suggest that they have more conserved RNA1 (<5% of nucleotide sequence variability) compared to RNA2 (up to 7% of variability) molecules. The highest nucleotide sequence divergence among PfGSV isolates and other cileviruses is in the genomic segment covering from the 5′-end of the RNA2 until the 5′-end of the open reading frame (ORF) *p61*, which includes the ORF *p15* and the intergenic region. This genomic stretch also harbors a novel orphan ORF encoding a 13 kDa protein presenting a cysteine-rich domain. High variability of 5′-end of the RNA2 in cileviruses is discussed in an evolutionary context assuming that they share putative common ancestors with unclassified arthropod-infecting single-strand positive RNA viruses, including mosquito-specific viruses of the group Negevirus (clades Nelorpivirus and Sandwavirus), and other viruses in the family *Kitaviridae.*

## Introduction

Passion fruit (*Passiflora* spp.) crops can be severely affected by viral infections that may cause up to 100% of production losses and limit their commercial expansion around the tropical and near-tropical regions of the world (Fischer and Rezende, 2008; Santos et al., 2015; Atukunda et al., 2018). South America supports the greatest collection of *Passiflora* spp. where Colombia and Brazil are considered as *Passiflora* diversity hot spots (Cerqueira-Silva et al., 2016). Several *Passiflora* spp. are well valued as human food, in the cosmetic industry, for the treatment of some human illnesses, and as ornamentals (Yockteng et al., 2011; Zeraik et al., 2011). In the period 2015–2017, the Brazilian passion fruit harvest represented approximately 65% of the worldwide production (Altendorf, 2018). Passion fruit woodiness disease, caused by a potyvirus, is the major disease affecting the passion fruit crop in that country (Nascimento et al., 2006; Rodrigues et al., 2015; de Oliveira Freitas et al., 2016).

Since the identification in the 1990s, two seemingly different viral diseases known as passion fruit green spot (PfGS) and passion fruit sudden death (PfSD) intermittently occur in passion fruit orchards in Brazil (Kitajima et al., 1997, 2003; Santos Filho et al., 1999). In PfGS disease, spots of approximately 5 mm showing brilliant green borders with or without a central necrotic dip develop on the peel of yellow mature fruits, and necrotic lesions appear on the stems. These stem wounds, which sometimes exhibit deep slits, may also coalesce and encircle the branch leading to the death of the distal end. In leaves, randomly distributed spots intersperse with uneven patches commonly observed alongside the veins. Usually, yellowish foliar lesions arising during the initial stages of the infection gradually turn green island-like spots on the senescent leaves. As a consequence of PfSD disease, affected plants display the major symptoms of PfGS, but they rapidly progress toward death, passing through abundant necrosis, branch death, and finally, the orchard’s collapse (Antonioli-Luizon et al., 2009).

Both PfGS and PfSD diseases are transmitted by false-spider mites of the genus *Brevipalpus* (Acari: *Tenuipalpidae*) (Kitajima et al., 2003). Experimental reproduction of PfGS symptoms on leaves and stems was achieved by transferring brevipalpus mites collected from affected field passion fruit plants onto healthy ones (Kitajima et al., 1997, 2003). In addition, the ubiquity of short, bacilliform virus particles (50–70 nm × 100–120 nm) in the cisternae of the endoplasmic reticulum, and viroplasms in the cytoplasm of the infected plant cells suggested the infection by putative cileviruses as their causal agents (Kitajima et al., 2003). From isolated viral dsRNA molecules from PfGS-affected passion fruit plants, a PCR-based molecular detection method allowed the specific detection of a virus tentatively named as passion fruit green spot virus (PfGSV) (Antonioli-Luizon, 2010). This approach revealed the prevalence of PfGSV in PfGS affected plants (Kitajima et al., 2003; Antonioli-Luizon et al., 2009). Amplicon-derived sequences (GenBank accession numbers HM002746 and HM002747) showed < 60% pairwise nucleotide sequence identity to the cilevirus citrus leprosis virus C (CiLV-C) (Antonioli-Luizon, 2010). However, despite the growing body of evidence, PfGSV has not been classified due to the lack of genome sequence data and, consequently, its unclear evolutionary history with other members of the genus *Cilevirus*.

Genus *Cilevirus*, family *Kitaviridae*^1^ has viruses with short bacilliform and enveloped particles that encapside two molecules of single-strand positive sense [ss(+)] RNA as genome (Freitas-Astúa et al., 2018). CiLV-C and citrus leprosis virus C2 (CiLV-C2) are the only recognized members of this genus (Locali-Fabris et al., 2006; Roy et al., 2013). Both CiLV-C and CiLV-C2 show a narrow range of natural hosts, produce non-systemic diseases and are persistently transmitted by *Brevipalpus yothersi* mites in a circulative manner (Bastianel et al., 2010; Roy et al., 2015; Ramos-González et al., 2016). RNA genomic molecules of CiLV-C, type-member of the genus, are 3′-polyadenylated. RNA1 molecule, of ∼9 kb, has two open reading frames (ORFs) that encode the RNA-dependent RNA polymerase (RdRp) and the putative 29 kDa capsid protein. The ∼5 kb RNA2 has three ORFs encoding proteins with unknown functions, i.e., the two taxonomically restricted ORFs *p61* and *p24*, and the orphan ORF *p15*, in addition to the *mp* that encodes the putative movement protein (MP). ORFs *p15* and *p61* are separated by a stretch of ∼1000 nts known as the intergenic region (IR). CiLV-C proteins can form homo- and heterodimers, and associate with plant cell membranes producing severe remodeling of the endoplasmic reticulum and the Golgi complex (Leastro et al., 2018).

Phylogenetic analyses including plant-infecting viruses of the families *Kitaviridae, Bromoviridae*, *Closteroviridae*, and *Virgaviridae*, reveal a distant but consistent relationship with an increasing number of arthropod-infecting viruses [ss(+)RNA non-segmented genomes] suggesting that they share a common ancestor (Li et al., 2015; Vasilakis and Tesh, 2015; Shi et al., 2016; Kondo et al., 2019; Vinokurov and Koloniuk, 2019). The genome of some of these viruses, e.g., members of the group Negevirus (proposed genera Nelorpivirus and Sandewavirus) and from the negevirus-like lineage have, besides a large ORF encoding the viral RdRp, the ORF2, and ORF3, which encode a putative glycoprotein and a small membrane-bound protein (Vasilakis et al., 2013; Nunes et al., 2017). These two proteins show structural features, e.g., SP24 (Pfam: 16504), transmembrane domains, signal peptides, which are conserved across their orthologs (P61 and P24 in CiLV-C) in viruses of the family *Kitaviridae* (Kuchibhatla et al., 2014).

In this current work, we describe the identification and genome sequence of viruses belonging to a tentative new species of cilevirus: passion fruit green spot virus (PfGSV). Analysis by high-throughput sequencing (HTS) and Sanger sequencing of RT-PCR amplicons from PfGS and PfSD diseased plants collected in distantly and economically important growing regions in Brazil indicated the ubiquity of PfGSV in these samples. Genome analysis of the viruses revealed the presence of a putative novel ORF in the RNA2 molecule, which lacks homologous sequences in both the genome of known cileviruses and any other documented nucleotide or amino acid sequence. In this regard, it might represent a new ORF that enlarges the list of orphan and taxonomically restricted genes only detected in members of the family *Kitaviridae* and related invertebrate-infecting viruses. In parallel, we revealed the genome sequences of two potyviruses found in mixed infections with PfGSV in two out of the three samples analyzed by HTS. Finally, supported by phylogenetic and ancestral trait reconstruction analyses we hypothesize the putative origin of kitaviruses and other unassigned nege-like viruses from arthropod-infecting common ancestors.

## Materials and Methods

### Plant Material

**FIGURE 1:**
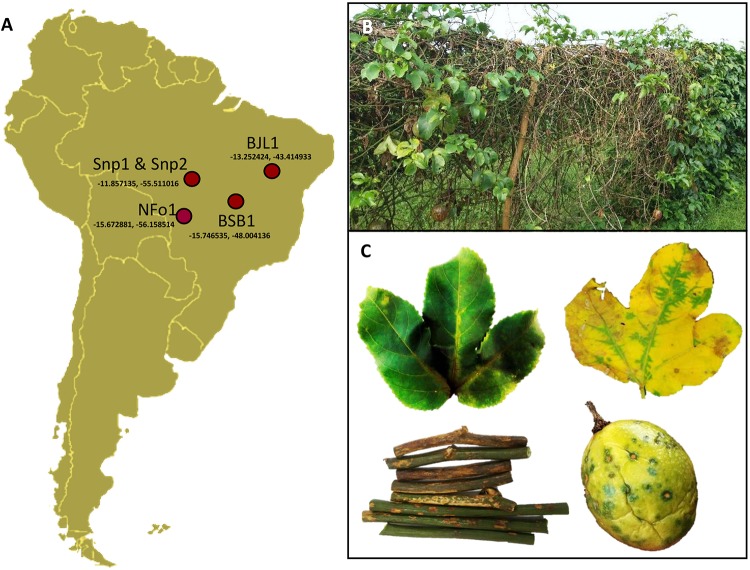
Symptoms of passion fruit green spot virus (PfGSV)-infected passion fruit plants. **(A)** Samples of diseased passion fruit plants (*Passiflora* spp.) were collected in four regions of Brazil. **(B)** Overview of a PfGSV-affected orchard in Sinop, MT, Brazil. **(C)** Details of chlorotic and necrotic symptoms on leaves, stems, and fruits. Note the yellow strips along the veins in green young leaves (left) that turn green areas in a yellow background covering the non-infected areas in the oldest leaves (right).

Passion fruit (*Passiflora* spp.) plants showing PfSD symptoms (green spots on fruits, necrotic lesions on stems, and chlorotic strips alongside the leaf veins) were collected in counties near Sinop (Snp) and Nova Fronteira (NFo), both in the State of Mato Grosso, in Central-North Brazil, in 2016 and 2018, respectively; and in Bom Jesus da Lapa (BJL), State of Bahia, in Northeastern Brazil, in 2008 (Figure 1). A sample from a passion fruit plant (*Passiflora* sp.) displaying PfGS symptoms was collected in Brasilia (BSB), Distrito Federal, in Central Brazil, in 2003. Samples and viral isolates were identified accordingly with their geographic origins, i.e., Snp1, Snp2, NFo1, BJL1, and BSB1. Straight-line distances between these towns range from 430 to 1396 km (Snp-NFo: 430 km, BSB-BJL: 578 km, Snp-BSB: 880 km, BSB-Snp 934 km, BJL-Snp: 1321 km, and BJL-NFo: 1396 km). Fragments of leaves, stems, and fruits were prefixed in a modified Karnovsky solution (Kitajima and Nome, 1999) for transmission electron microscopy (TEM) analyses as previously described (Chabi-Jesus et al., 2018). Other fragments from the symptomatic samples were conserved at −80°C until RNA extraction.

### Detection of Passion Fruit Green Spot Virus

Total RNA was extracted from approximately 100 mg of plant samples using TRIzol^®^ Reagent and following the manufacturer’s recommendation (Life Technologies, Foster City, CA, United States). Approximately 500 ng of the RNA templates were used to obtain the cDNA using random hexamer primers and GoScript^TM^ Reverse Transcriptase Kit as described by the manufacturer (Promega, Madison, WI, United States). Three microliters of each cDNA solution were tested by PCR using three pairs of primers for the detection of PfGSV: RNA1 C13F: 5′-ATTCATGCGTTTCACGGTTA-3′, C13R: 5′-CGAATGCCTCTGACACAACT-3′, and PfGSV: RNA2 C6F: 5′- CGATATTTGATCAATCCGTT-3′, C6R: 5′-CACCTTAAAATT CGAGGGTT-3′, C8F: 5′-TTCATCGCAAGTTCGTATACCT-3′, and C8R: 5′-CTGTTGTGCCAAATCATCAA-3′ (Antonioli-Luizon, 2010). Amplicons were separated on 1% agarose gels in 1X Tris-acetate-ethylenediaminetetraacetic acid (TAE) and visualized with ethidium bromide (0.1 μg/mL). Some amplicons were further purified using Wizard^®^ SV Gel and PCR Clean-Up System (Promega, Madison, WI, United States) and sequenced by the Sanger method.

### Sequencing and *in silico* Assembly of Viral Genomes

RNA extracts independently obtained from samples Snp1 (2016), BSB1 and BJL1 were further purified using RNeasy Mini Kit (QIAGEN, Venlo, Netherlands). Quantification and estimation of the A260/A280 ratio were carried out using a NanoDrop ND-8000 micro-spectrophotometer (Thermo Scientific, Waltham, MA, United States). Five hundred nanograms of each final extract were sent to the Animal Biotech Laboratory at Escola Superior de Agricultura Luiz de Queiroz, University of São Paulo (Piracicaba, Brazil) for HTS using HiSeq 2500 Technology (2 × 150 nt paired-end reads) (Illumina, San Diego, CA, United States). Preparation of the HTS libraries was carried out as previously defined (Ramos-González et al., 2017). Read assembly up to the generation of the viral-sequence-containing contigs were done by using both SPAdes (Bankevich et al., 2012) available in Geneious software package version 11.1.4 (Kearse et al., 2012) and Trinity (Haas et al., 2013). Contigs were annotated using BlastX, implemented in the Geneious software, against both viral genome database^2^ and a customized local database including the sequences of kitavirids: cileviruses, blunerviruses (Quito-Avila et al., 2013; Hao et al., 2018), and the higrevirus hibiscus chlorotic green spot virus 2 (HGSV-2) (Melzer et al., 2013a). Those contigs producing the best E-value score (∼0) with the cilevirus RNA1 and RNA2 molecules of either CiLV-C (GenBank accession numbers DQ352194 and DQ352195) or CiLV-C2 (JX000024 and JX000025) were selected. A set of 32 primers that generate overlapping amplicons were designed with the program Primer3 (Untergasser et al., 2012) and taking as reference the largest contigs corresponding to each genomic segment of the Snp1 viral isolate (Supplementary Table S1). These primers and the total RNA of the sample Snp1 were subsequently used to validate the HTS and to obtain the sequences of the 5′-ends of both genomic molecules of the viral isolate Snp1 using a RACE SMARTer^®^ RACE 5′/3′ Kit (Clontech Laboratories, Mountain View, CA, United States). For RACE analyses, amplicons were purified, ligated in pGEM-T-easy vector (Promega, Madison, WI, United States), and further sequenced with universal primer pairs M13F/M13R by the Sanger method (Instituto Biológico, SP, Brazil). At least five clones per each type of amplicons were sequenced. Snp1-specific primers were also used to generate the near-complete genome of the viruses in samples Snp2, collected in 2018, and NFo1. Sanger method-generated sequences of the amplicons were assembled using CAP3 assembly (Huang and Madan, 1999) implemented in Geneious (Kearse et al., 2012). Sequences of the assembled genomes were submitted to the GenBank database and assigned to the following GenBank accession numbers: PfGSV_Snp1 RNA1: MK804171 and RNA2: MK804172, PfGSV_BSB1 RNA1 MK804173: and RNA2 MK804174, PfGSV_BJL1 RNA1: MK804175 and RNA2: MK804176, PfGSV_Snp2 RNA1: MN746810 and RNA2: MN746811, and PfGSV_NFo1 RNA1: MN746812 and RNA2: MN746813.

### Northern Blot Assays

Ten micrograms of denatured RNA from both PfGSV_Snp1-infected and healthy passion fruit plants were separated in 1% agarose gel containing formaldehyde. The assay also included RNA extracts from CiLV-C-infected and healthy sweet orange plants. Non-radioactive probes were generated using the PCR DIG-labeling kit (Roche Diagnostics, Mannheim, Germany) according to the manufacturer’s instructions. Each probe was obtained in independent reactions adding 1 ng of DNA fragments corresponding to each PfGSV_Snp1 or CiLV-C_CRD ORF (*p29* or *p24*) and the appropriate primer pairs. DNA labeling was verified comparing the amplicon sizes of DIG PCR products with the unlabeled DNA fragments. Hybridization and detection were carried out as recommended by the Dig Luminescent Detection Kit manual (Roche Diagnostics, Mannheim, Germany). The presence of bands in the blots was revealed using the BCIP-NBT substrate.

### Genome, Protein, and Phylogenetic Analyses

Viral ORFs were identified *in silico* by the ORF finder^3^. Conserved domain architecture, presence of signal peptide, and predictions of transmembrane helices, subcellular localization and isoelectric points of viral proteins were done by using SPARCLE^4^ (Marchler-Bauer et al., 2017), Pfam v 32.0 EMBL-EBI^5^, SignalP 5.0 (Almagro Armenteros et al., 2019), TMHMM Server 2.0^6^ (Sonnhammer et al., 1998), Deeploc v1 (Almagro Armenteros et al., 2017), and ExPASy Compute pI/Mw tool^7^. The presence of coiled-coil regions was detected using the neural network-based tool DeepCoil (Ludwiczak et al., 2019). Database searching for remote protein homology detection was carried out using the MPI Bioinformatics Toolkit (HHblits, HHpred, and HMMER)^8^ (Hildebrand et al., 2009; Finn et al., 2011; Remmert et al., 2012; Zimmermann et al., 2018). Nucleotide and predicted amino acid sequences were aligned using the CLUSTAL algorithm (Chenna et al., 2003) implemented in MEGA X version 10.0.5 (Kumar et al., 2018) and MAFTT (Katoh and Standley, 2013), respectively. Additionally, alignments and predicted secondary structures of deduced amino acid sequences of the putative viral proteins were obtained using PROMALS (PROfile Multiple Alignment with predicted Local Structure) (Pei and Grishin, 2007) and PRALINE (Profile ALIgNmEnt) (Bawono and Heringa, 2014).

For phylogenetic analyses, besides the sequences from known kitaviruses and negeviruses, others from non-classified ss(+)RNA viruses were retrieved from GenBank after a BLAST search (cut-off E-value < e^–10^) using PfGSV and Negev virus (NC_030294.1) sequences as the query. Some plant-infecting viruses of the family *Virgaviridae* were included in the study as an external group. Phylogenetic informative regions of the multiple sequence alignments (MSAs) were selected using BMGE software (Criscuolo and Gribaldo, 2010) implemented in https://ngphylogeny.fr (Lemoine et al., 2019). Substitution models with the lower Bayesian information criterion scores for each MSA were determined using MEGA X version 10.0.5 (Kumar et al., 2018). For the MSA of the RdRp proteins, the sequences of the domains methyltransferase and helicase, and RdRp encoded by the RNA1 and RNA2 molecules, respectively, of the blunerviruses blueberry necrotic ring blotch virus strains Giorgia and RL (BNRBV_Giorgia and BNRBV_RL, respectively) and tea plant necrotic ring blotch virus (TPNRBV) were concatenated as previously studied (Quito-Avila et al., 2013). Phylogenetic relationships were inferred by using MrBayes v3.2.6 implemented in Geneious prime software package version 2019.2.3 (Kearse et al., 2012). MCMC convergence was obtained for four independent runs of 6 million generations, which were sufficient to obtain a proper sample for the posterior probability, assessed by effective sample sizes (ESSs) above 200. The posterior probabilities of the clades were determined by a 50% majority consensus of the trees retained. Trees were edited and visualized using Interactive Tree Of Life (iTOL) v4 (Letunic and Bork, 2019).

Ancestral trait reconstruction was carried out using the Bayesian Binary MCMC (BBM) analysis implemented in RASP v4.2 (Yu et al., 2019). The 14,142 post-burn-in trees from the RdRp Bayesian Inference analysis using MrBAYES were input into RASP to estimate the probabilities of ancestral hots at each node on the condensed tree generated by RASP. Hosts were coded as Arthropods, Plants, Anemones, and Nematodes. MCMC chains were run simultaneously for 5,000,000 generations and the reconstructed state was sampled every 1,000 generations. The fixed model JC + G (Jukes-Cantor + Gamma) was used for the BBM analysis.

## Results

### Identification of Passion Fruit Green Spot Virus in Symptomatic Passion Fruit Plants

**FIGURE 2:**
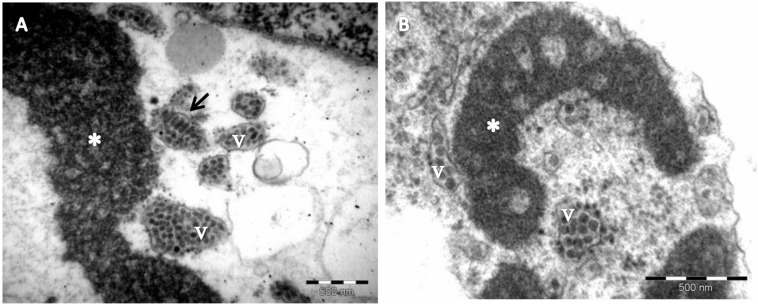
Electron micrograph of passion fruit green spot virus (PfGSV)-infected passion fruit leaves. **(A,B)** Micrographs of thin sections of tissues from green patches on senescent passion fruit leaves, collected in commercial fields near Sinop, State of Mato Grosso, Brazil. Large, electron-dense and vacuolated inclusions (viroplasms) (^∗^) are present in the cytoplasm of parenchymal cells. Short, bacilliform particles, presumed PfGSV virions, can be seen accumulating within cisternae of the endoplasmic reticulum, either in cross- (v) or longitudinal (arrow) sections.

The occurrence of PfGSV was assessed in passion fruit plants showing symptoms ascribed to the PfGS and PfSD diseases, i.e., circular necrotic spots on fruits, and chlorotic and necrotic lesions on their leaves and stems (Figures 1B,C). The presence of the suspected virus was first verified by RT-PCR assays using the primer pairs C6, C8, and C13 (Antonioli-Luizon, 2010). All the samples tested positive with at least one of the primer pairs used, and amplicons of the expected sizes were visualized (Supplementary Figure S1). However, the plant organ used for the RNA extractions apparently affected the efficiency of these tests. Only a quarter of the symptomatic stem samples were RT-PCR positive whereas 100% of the leaf samples were positive (Supplementary Figure S1). Amplicons obtained using the C6 and C13 primer pairs showed 98–99.2% nucleotide sequence identity with fragments of the PfGSV genome (GenBank accession numbers HM002746 and HM002747). In addition to the molecular detection, virus-like particles with a bacilliform shape of approximately 50 nm width × 100 nm length were observed in the cytoplasm of parenchymal cells of the passion fruit leaves of the sample Snp1 by TEM (Figure 2). Presumed viral particles were always found in the lumen of cytosolic vesicles whereas electron-dense and vacuolar viroplasms occupied large areas of the cytoplasm.

### Passion Fruit Green Spot Virus Genome Resembles That of Known Cileviruses

Genome sequences of five isolates of PfGSV were obtained from distinct diseased plants. Isolates Snp1, BSB1, and BJL1 were assembled from HTS reads and Snp2 and NFo1 by Sanger sequencing of overlapping amplicons obtained by RT-PCR.

HTS libraries from the BSB1 and BJL1 isolates contained the highest PfGSV RNA matched reads with 3.9 and 4.2%, respectively, out of the total recovery reads (Supplementary Table S2). Snp1’s library contained the lowest percentage of viral-derived reads (2.8%), probably indicating a lower viral load in that sample. Regardless of the origin of libraries, the numbers of reads corresponding to the RNA2 molecules surpassed those derived from the RNA1 molecules. The minimum value of mean coverage per viral base reached 9,369X and it corresponded to the RNA1 molecule of the Snp1 isolate. The 5′-ends of the RNA1 and RNA2 molecules of the Snp1 isolate were obtained by RACE.

Excluding the poly-A tails at their 3′-end, the genome of PfGSV_Snp1 consists of 13,523 splits into two molecules designated as RNA1 and RNA2 with 8,740 and 4,783 nts, respectively (accession numbers MK804171 and MK804172). Near-complete RNA1 molecules of the isolates BSB1 (MK804173), BJL1 (MK804175), Snp2 (MN746810), and NFo1 (MN746812) include 8,694; 8,717; 8,541 and 8,554 nts; whereas their near-complete RNA2 consist of 4,752; 4,769, 4,578 and 4,545 nts (MK804174, MK804176, MN746811, and MN746813), respectively. Shorter molecules in the isolates BSB1, BJL1, Snp2, and NFo1 may be a direct consequence of the strategies deployed during their sequencing. Despite this, when compared with PfGSV_Snp1 molecules, more than 95% of the genome of each isolate was recovered.

Pairwise comparisons of the genomic molecules of the isolate Snp1 with those from Snp2, NFo1, and BSB1 revealed a high nucleotide sequence identity (RNA1 > 99.1% and RNA2 > 97.3), whilst with the BJL1 isolate the values were slightly lower (RNA1: 95.5% and RNA2: 93.3%) (Table 1). Due to the high nucleotide sequence identity between the PfGSV isolates, Snp1 will be considered the type variant of PfGSV. Therefore, further analysis will be focused on Snp1, and also on isolates BSB1, and BJL1, which collectively were collected from distant geographic regions and whose genomes were more extensively obtained.

**TABLE 1:** Nucleotide (nt) and deduced amino acid (aa) identities of passion fruit green spot virus (PfGSV).

**PfGSV_Snp1**	**PfGSV_BSB1**	**PfGSV_BJL1**	**PfGSV_Snp2**	**PfGSV_NFo1**	**CiLV-C_CRD**	**CiLV-C_SJP**	**CiLV-C2_Co**	**CiLV-C2_Hw**
	nt/aa^a^	nt/aa	nt/aa	nt/aa	nt/aa	nt/aa	nt/aa	nt/aa
**RNA1**	99.3/–	95.5/–	99.3/–	99.1/–	60.0/–	59.8/–	71.5/–	**72.0**/–
*RdRp*	99.4/99.8	95.1/99.5	99.3/99.7	99.1/99.7	61.1/57.8	60.8/57.8	72.5/81.0	**73.1**/**81.0**
*p29*	99.1/99.2	97.5/100.0	99.4/100	99.4/100	48.8/32.7	49.9/31.7	**62.8**/**63.4**	62.0/60.2
**RNA2**	99.2/–	93.3/–	97.3/–	97.3/–	52.6/–	53.0/–	**63.7**/–	62.8/–
*p15*	99.7/99.2	87.0/83.3	96.4/95.4	93.9/84.7	48.0/14.1	46.7/14.1	**60.3**/**56.7**	57.2/55.6
*p13/p11*	99.7/100.0	88.8/36.9	98.8/98.2	99.7/99.1	–	–	**–**	**–**
IR*^b^*	99.0/–	91.3/–	98.7/–	98.6/–	37.6/–	37.4/–	**44.8/–**	**44.8/–**
*p61*	99.1/99.4	92.1/94.8	96.8/98.0	98.7/98.9	50.0/31.6	51.1/31.8	63.7/59.3	**63.9**/**60.4**
*mp*	99.4/99.7	97.6/99.3	97.5/99.7	96.7/99.3	56.2/51.2	56.3/48.8	**71.5**/**74.3**	68.0/70.5
*p24*	99.7/100.0	96.5/100.0	96.8/99.5	95.7/99.5	63.8/60.8	64.6/59.6	**78.2**/**88.0**	77.2/86.1

*Comparisons between PfGSV_Snp1 and the isolates BSB1, BJL1, Snp2, NFo1, and the citrus-infecting cileviruses. The highest values are highlighted in bold. ^*a*^Percentage of nucleotide sequence identity/amino acid sequence identity. ^*b*^IR: Intergenic region as identified in CiLV-C. This region encompasses the nucleotide sequences between 3′-end of *p15* ORF and 5′-end of *p61* ORF in the RNA2 molecule.*

**FIGURE 3:**
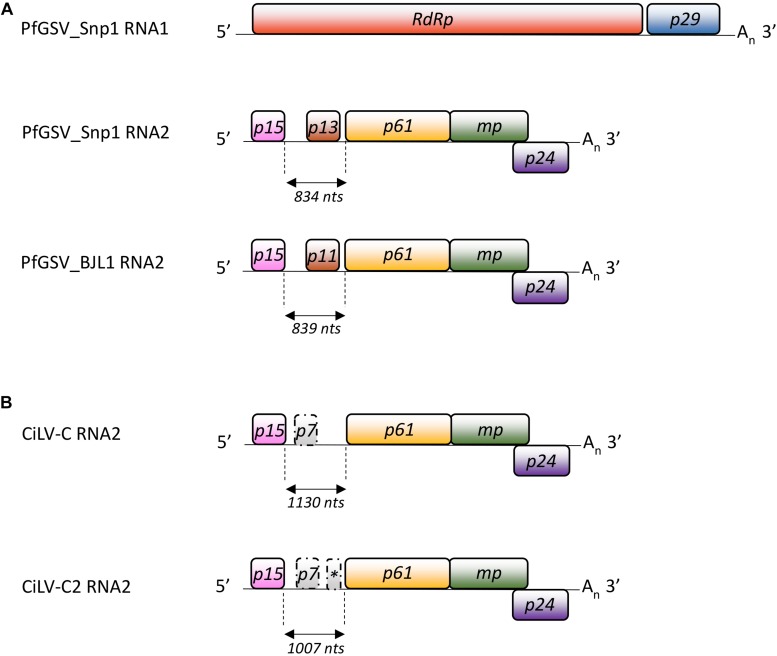
Genomic structure of passion fruit green spot virus. Graphic representation of **(A)** the PfGSV-Snp1 genome and its comparisons with the RNA2 molecules from PfGSV_BJL1 and **(B)** the cileviruses CiLV-C and CiLV-C2. Solid bars indicate the ORFs. *RdRp*: RNA-dependent RNA polymerase; *p29*: putative coat protein; *MP*: putative movement protein; *p15*, *p13*, *p11*, *p61*, and *p24*: proteins with unknown functions. ORFs *p7* and that identified with an asterisk in the IR of CiLV-C and CiLV-C2 have been poorly described. A_n_: poly(A) tail.

Seven putative ORFs with more than 200 nts were detected in the PfGSV genomes, two in the RNA1 and five in the RNA2 (Figure 3A). ORFs *p15* and *p13*, in the 5′-end of the RNA2, encompass the largest discrepancies among the coding nucleotide sequences of BJL1 with the other isolates. Comparisons of PfGSV_Snp1 with known cileviruses revealed the highest nucleotide and deduced amino acid sequences identities with CiLV-C2 isolates Hw and Co (Table 1). At the genomic level, the highest identity values corresponded to RNA1 molecules (∼72.0%); while the RNA2 segments are more divergent (∼64.0%), even though they harbor the *p24* ORF that is the most conserved ORF across cileviruses. In the comparisons with the CiLV-C2 isolates, P29, P15, and P61 proteins accounted for the lower levels of amino acid identities. In general, regardless of the evaluated gene, deduced protein or virus, the lowest values of identity always corresponded to the comparisons between PfGSV and CiLV-C.

From the seven putative polypeptides encoded by the genome of PfGSV, the RdRp (RNA1) is the largest one, encoding a 2506 aa protein (285.9 kDa). In PfGSV_Snp1 this protein shows the viral methyltransferase domain (Pfam: PF01660) located near to the NH_2_-terminus of the protein. RdRp also includes the viral helicase_1 (Pfam: PF01443) and the RdRP_2 (Pfam: PF00978) domains, both localized in the second half of the protein, being the RdRP_2 motif the nearest to the COOH-terminus. P29_Snp1 (RNA1: 258 aa; 26.6 kDa), the putative coat protein, although with a relatively low level of amino acid identity with isolates of the citrus infecting-cileviruses (<33% with CiLV-C and <65% with CiLV-C2, Table 1), shows stretches of highly conserved residues among cileviruses. Eighteen out of the first 32 amino acids at the NH_2_-terminus of these proteins are conserved, while five other positions display residues with similar biochemical characteristics (Supplementary Figure S2). Alignment of P15 (RNA2: 131 aa; 14.8 kDa) from the PfGSV isolates with those from cileviruses displays 20 invariable amino acid residues. Some of them are conserved Cys residues likely essential for the P15 functioning, as previously described for CiLV-C (Ramos-González et al., 2016). Nonetheless, P15_Snp1 has an amino acid identity of < 15% in comparison with its cognates from CiLV-C strains, being, in general, the most divergent protein among cileviruses (Table 1).

The largest protein encoded by the RNA2 is the putative glycoprotein P61 (543 aa; 61.0 kDa). *In silico* analyses of P61_Snp1 revealed the presence of a signal peptide, which is likely cleaved between the positions 25 and 26, residues Ser-Lys-Gly/Arg-Phe. Besides three putative N-glycosylation sites at positions 249, 310, 340 in the mature peptide, P61_Snp1 also shows two adjacent transmembrane domains located near the COOH-terminus of the protein (positions 442–466 and 471–498). Similar structural features are conserved through the P61 of the isolates BJL1 and BSB1. MP_Snp1 (RNA2: 295 aa; 32.6 kDa) analysis revealed the presence of the 3A motif (Pfam: 00803) involved in the cell-to-cell movement of some plant-infecting viruses of the 30K superfamily (Melcher, 2000). This protein also has the typical “D motif”, which occurs in the MPs encoded by a diverse group of plant viruses (Mushegian and Elena, 2015).

P24 protein is the most conserved among those encoded by cileviruses. P24_Snp1 (207 aa; 23.8 kDa) has 88.0 and 60.8% amino acids sequence identity, respectively, with its cognate proteins from CiLV-C2_Co and CiLV-C_CRD (Table 1). All the studied isolates of PfGSV has the SP24 motif (Pfam: 16504), which, besides in cileviruses, is found in several arthropod-infecting viruses such as chroparaviruses, negeviruses, negevirus-like viruses, and in plant-infecting viruses of the genera *Higrevirus* and *Blunervirus* (Kuchibhatla et al., 2014; Nunes et al., 2017; Viljakainen et al., 2018). The alignment of P24 proteins from cileviruses reveals a less conserved NH_2_-terminus (approximately first 30 aa) that in the case of P24_Snp1 also shows a high density of positively charged residues (five Arg and three Lys) (Supplementary Figure S3). P24_Snp1 has several helices that might determine four transmembrane domains: TM1 (residues 46–62), TM2 (82–100), TM3 (112–130), and TM4 (150–169). Particularly, residues comprised between positions 48 and 59 are predicted to form a coiled-coil domain (Supplementary Figures S3C,D).

### The Intergenic Region in the RNA2 From PfGSV Is Shorter Than Those of Known Cileviruses and Harbors a Novel Orphan ORF

In their RNA2 molecules, between the ORFs *p15* and *p61*, the cileviruses CiLV-C and CiLV-C2 have a stretch of non-coding nucleotide sequences known as the IRs. In the PfGSV strains Snp1, BJL1, BSB1, these regions are 834, 839, and 834 nts in length, respectively (Figure 3A). Following the trend observed in the comparisons among the ORFs and deduced proteins encoded by the PfGSV genomes (Table 1), the nucleotide sequence identity between IRs from the isolates Snp1 and BSB1 are higher (99.0%) than that from the isolates Snp1 and BJL1 (91.3%). When compared with other cileviruses, the stretch of nucleotide sequences between *p15* and *p61* in PfGSV isolates are 17–35% shorter than those displayed in CiLV-C2 (strains Co: 1007 nts, Hw: 1078 nts, and Fla: 1048 nts), and 25–43% smaller than those in CiLV-C (strains CRD: 1130 nts and SJP: 1135 nts) (Figure 3B). The alignments of regions between the ORFs *p15* and *p61* from PfGSV, CiLV-C and CiLV-C2 show, at best, 45% nucleotide sequence identity (Table 1).

Besides the relatively low sequence identities, the stretch between the ORFs *p15* and *p61* in the PfGSV isolates Snp1, Snp2, NFo1, and BSB1 display an ORF (*p13*) with 330 nts that encodes a putative polypeptide of 110 residues, highly conserved, and ∼13 kDa (Figure 3A) (Table 1). It is noteworthy that due to the existence of the ORF *p13* the use of the term IR to designate the stretch of nucleotide sequences between *p15* and *p61* in PfGSV, at least conceptually, is no longer conceivable.

**FIGURE 4:**
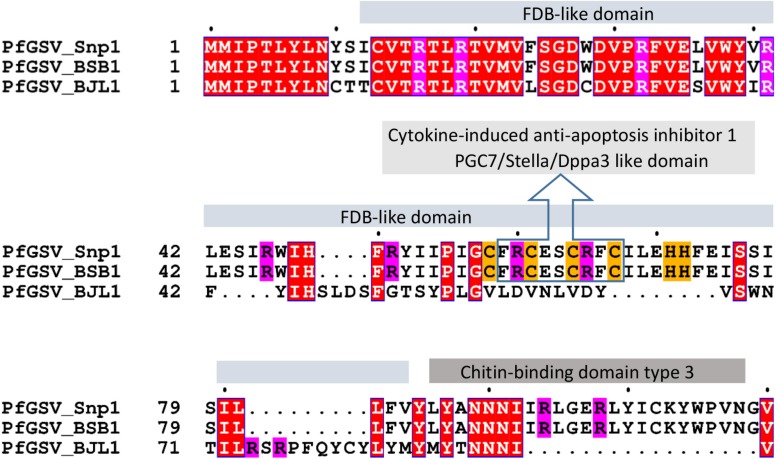
Deduced amino acid sequence alignment of proteins P13 and P11 from passion fruit green spot virus isolates Snp1, BSB1, and BJL1. Solids boxes on top of alignments label domains with remote identity in proteins P13, as identified by *HHblits*, *HHpred*, and *Smart* tools. White letters represent conserved amino acids across the three proteins. Magenta and mustard backgrounds highlight arginine (R), cysteine (C), and histidine (H) residues.

P13 protein has no apparent homology with any protein available in public databases. A thorough analysis using the *HHblits*, *HHpred*, and *Smart* tools revealed amino acid motifs that may be essential for the understanding of the putative origin and function of this protein. P13, estimated pI: 8.59, displays 11 positively charged residues (10 Arg + 1 Lys) and four regularly distributed Cys residues in a stretch of 15 amino acids that also contains two His residues: **Cys**_58_-Phe-Arg-**Cys**-Glu-Ser-**Cys**-Arg-Phe-**Cys**-Ile-Leu-Glu-**His**-**His**_72_ (Figure 4). This sequence shows a slight identity with motifs identified in the cytokine-induced anti-apoptosis inhibitor 1 (CIAPIN1, PF05093), and PGC7/Stella/Dppa3-like domain (PGC7_Stella, PF15549). The Cys-rich motif is embedded within a larger region that, according to *Smart*, shows a remote identity with the FDB domain found in FBox and BRCT domain-containing plant proteins (Doerks et al., 2002). A sequence of 26 residues in the COOH-terminal region of P13 shows a moderate identity with the Chitin-binding domain type 3 (InterPro IPR003610). In PfGSV_BJL1, the locus of its putative ORF *p13* is shorter. With only 282 nts, it encodes a smaller protein of 94 aa, ∼11.1 kDa, that shares almost the same sequence over the first 40 residues with P13_Snp1 (Figure 4).

### The Pattern of Subgenomic RNAs From PfGSV Resembles That Resultant From CiLV-C Transcription

Total RNA extract from the PfGSV_Snp1-infected plant was analyzed by Northern blot assay. The test also included RNA extract from CiLV-C-infected sweet orange leaves whose detected bands were used as molecular weight markers. Hybridizations were carried out using probes derived from the ORFs *p29* and *p24* of each virus. The identity of bands in each profile was assigned after a comparison between our results and those shown in a Northern blot analysis of CiLV-C previously obtained (Pascon et al., 2006).

**FIGURE 5:**
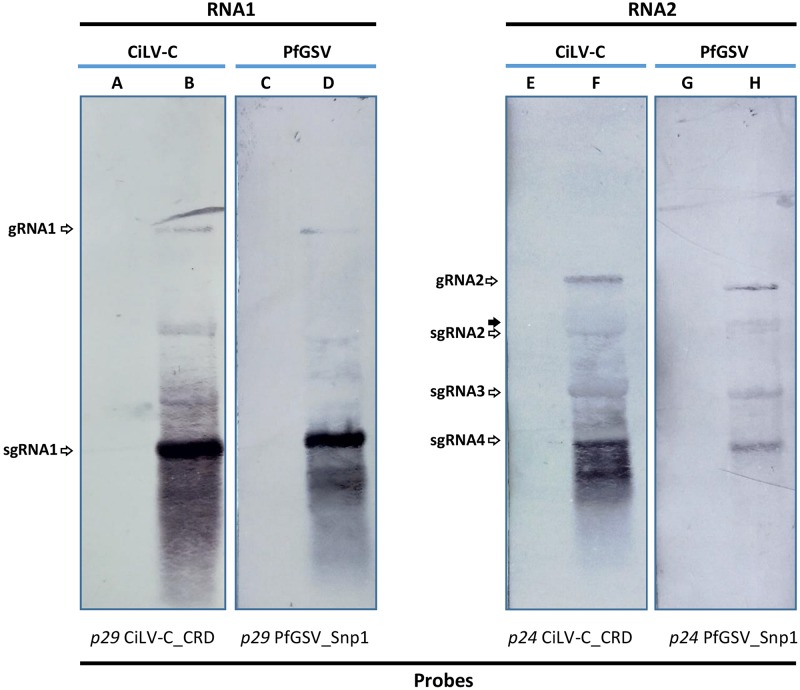
Passion fruit green spot virus RNA’s profile in infected plants. Northern blot hybridization using 10 μg of total RNA extracts from PfGSV-infected passion fruit plant **(D,H)**, which was compared with that of CiLV-C in sweet orange plant **(B,F)**. Digoxigenine (DIG-11-dUTP)- labeled probes were individually generated by PCR using specific primers for *p29* and *p24* ORFs from CiLV-C isolate CRD and PfGSV isolate Snp1. In lanes **A** and **C**, and **E** and **G** 10 μg of total RNA extracts from healthy sweet orange and passion fruit plants were assayed, respectively. The solid arrow indicates a band in the lane H that may correspond to sgRNA molecules for the putative expression of P13 protein.

Two bands were identified in the blots corresponding to the RNA1: gRNA1 and sgRNA1 (Figure 5). Relative amounts of these bands indicated much more accumulation of that corresponding to the sgRNA1, which harbors the ORF *p29* according to the probe used in this test. In blots hybridized with the *p24*-derived probes, besides the band of the gRNA2, three other bands were identified as sgRNA2, sgRNA3, and sgRNA4. sgRNA4, the most abundant and with the lowest molecular weight of the three bands, comprises the ORF *p24* region. sgRNA2 and sgRNA3 likely act as the mRNA for the expression of the ORFs *p61* and *mp*, respectively. New hybridizations using an ORF *p13* -specific probe need to be carried out to confirm whether a weak band with a molecular weight slightly higher than, and as abundant as, the sgRNA2 band in the PfGSV blots is the sgRNA from which the P13 protein may be expressed (Figure 5, lane H). No signal was detected in any of the lanes where RNA extracts from healthy plants were run (lanes A, C, E, and G). Other bands that remained unidentified seem to be the results of either partial degradation or defective products derived from the viral replication or transcriptional processes.

### Combined Phylogenetic and Ancestral Reconstruction Analyses Suggest That Direct Ancestors of Kitavirids Likely Colonized Arthropods

**FIGURE 6:**
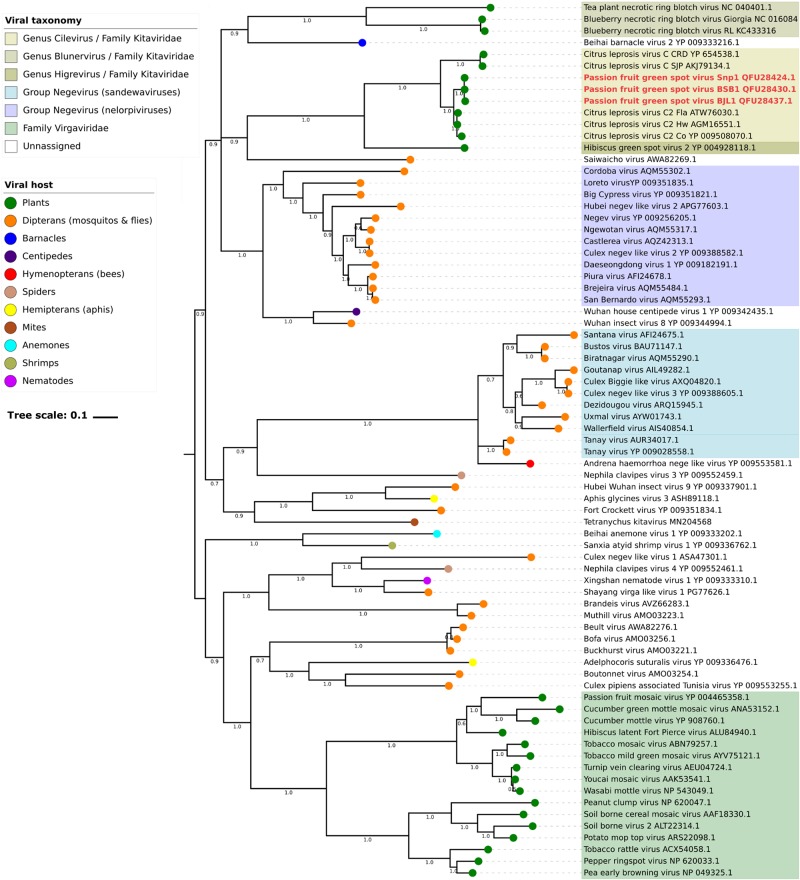
Molecular phylogenetic analysis of plant-infecting viruses of the family *Kitaviridae*. The midpoint-rooted Bayesian maximum clade credibility tree was inferred using a Markov Chain Monte Carlo (MCMC) of 6 million generations using the amino acid sequences of RNA dependent RNA polymerase (RdRp) of members of the family *Kitaviridae* (genera *Cilevirus*, *Higrevirus*, and *Blunervirus*), arthropod-infecting viruses, and viruses of the family *Virgaviridae*. Dataset included 443 positions and its evolutionary history was inferred based on the model LG + G + I + F (Le and Gascuel, 2008). Figures next to nodes indicate values of posterior probability branch support. Scale bar indicates the average number of amino acid substitutions per site. Passion fruit green spot virus sequences are highlighted in red.

Phylogenetic analyses of the RdRp and MP sequences revealed a closer relationship between PfGSV and strains of CiLV-C2, CiLV-C, and, although to a lesser extent, with other viruses in the family *Kitaviridae* (Figure 6 and Supplementary Figure S4). In the RdRp tree, viruses of the genera *Cilevirus* and *Higrevirus* shared the same clade with Saiwaicho virus, an unassigned monopartite virus found in *Drosophila suzukii* (Medd et al., 2018), while blunerviruses divided their clade with Beihai barnacle virus 2, an unassigned monopartite found infecting a crustacean (Shi et al., 2016). These two clades besides the clade comprising nelorpiviruses and two other unassigned ss(+)RNA viruses also found in arthropods, partook a polytomy, suggest either simultaneous speciation events or, more likely, absence of enough data to figure out how those lineages are related. Regardless of this, the inferred phylogenetic tree based on RdRp suggested the probable existence of common ancestors between plant-infecting kitaviruses and a number of arthropod-infecting viruses. In the MP tree, which as expected only included plant-infected viruses, a closer phylogenetic relationship was observed between cileviruses, then with the blunerviruses, and finally with the furoviruses oat golden stripe virus and sorghum chlorotic spot virus (Supplementary Figure S4).

**FIGURE 7:**
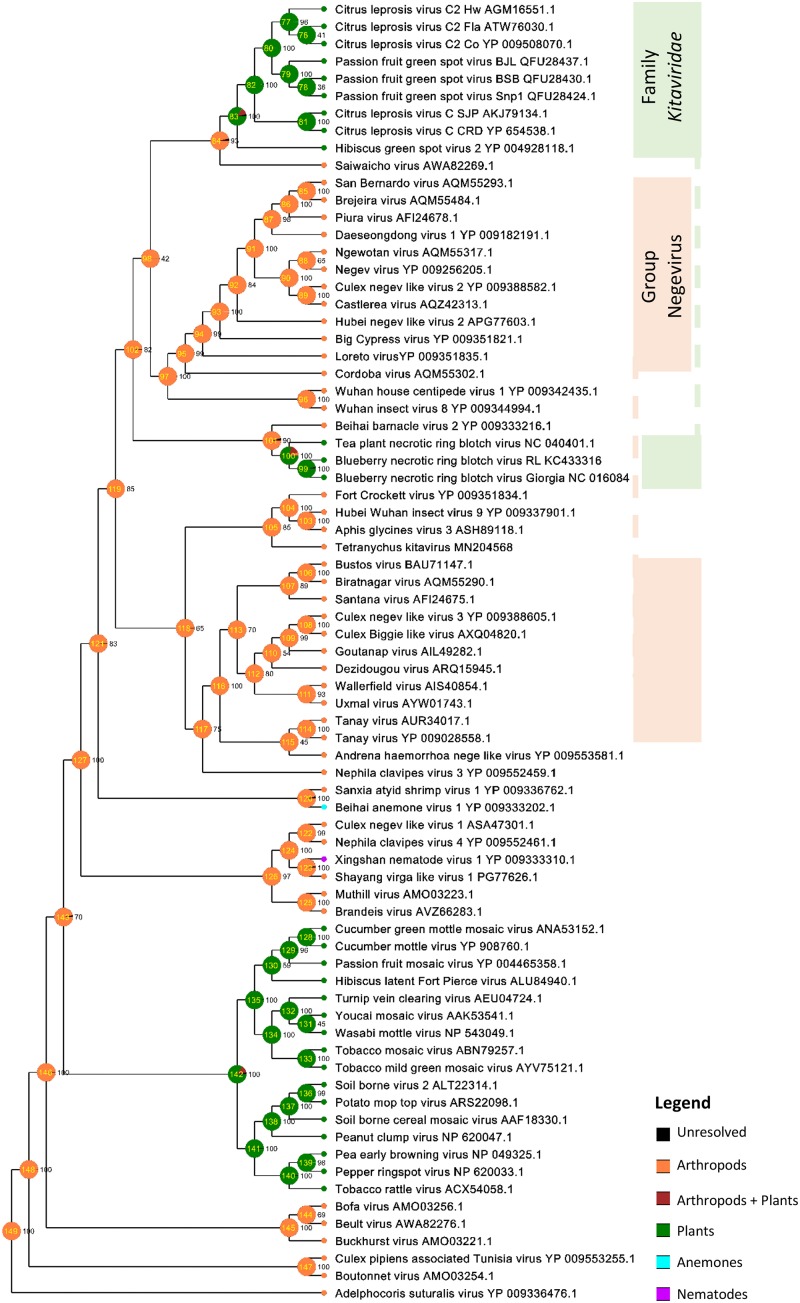
Ancestral state reconstructions performed by the Bayesian Binary MCMC analysis as implemented in RASP v4.2 using the RdRp MrBayes rooted tree. Pie charts at each node represent ancestral host estimations. Each node is internally identified with a number and the figures immediately at their right indicate their frequency in the consensus tree. The probabilities of particular hosts in major nodes are as follows: Node 84 – Arthropods (Arth) 96.15%, Arth + Plants (P) 3.40%, P 0.26%; Node 101– Arth 95.89%, Arth + P 3.55%, P 0.31%; Node 102 – Arth 99.08%, Arth + P 0.80%.

Ancestral trait reconstruction analysis based on the consensus RdRp phylogenetic tree indicated the likely hosts of most recently ancestors of kitaviruses (Figure 7). Nodes 84 and 101, which represent the common ancestors between viruses of the genera *Cilevirus*-*Higrevirus* and *Blunervirus*, respectively, with their closest arthropods-infecting relatives, are supported by more than 90% of trees, and they most likely infected arthropods (95%), as inferred by the RASP analysis. Similarly, the analysis of node 102, representing the most recent common ancestor of nelorpiviruses (group Negevirus) and kitaviruses, also indicated with a high probability (>99%) an arthropod-infecting virus. Node 98, although represented in the topology of the consensus tree, is not taken into account due to its relatively low support value (42%).

### Mixed Infections of Potyviruses and PfGSV in Passion Fruit Plants Affected by PfGS Disease

The near-complete genome sequences of two potyviruses, soybean mosaic virus (SMV) and cowpea aphid-borne virus (CABMV), were assembled from reads obtained from the analyses of the samples collected in Brasilia and Bom Jesus da Lapa, respectively. The genome of SMV_BSB1 (GB accession number MN124783) comprises 9,510 nts and shows the highest nucleotide sequence identity (97.7%) with SMV strain G4 (FJ640979) (Seo et al., 2009). Translated polyprotein of SMV_BSB1, 3,069 residues, shares 97.85% amino acid sequence identity with the SMV_Gulupa found infecting passion fruit plants in Colombia (Jaramillo-Mesa et al., 2018). The genome of CABMV_BJL1 (GB accession number MN124782) consists of 9,936 nts and encodes a polyprotein of 3,182 aa. Comparisons of CABMV_Snp1 with known potyviruses revealed both the best nucleotide (88.61%) and deduced amino acid (92.14%) sequence identities with CABMV_MG-Avr (HQ880243) (Barros et al., 2011). In the analysis of the HTS library obtained from the sample collected in Sinop, virus sequences other than that from PfGSV were not detected.

## Discussion

We have revealed the genome of five novel bipartite viruses found in passion fruit plants affected by either passion fruit green spot (PfGS) or passion fruit sudden death (PfSD) diseases. Our results not only complement previous works that suggested the presence of cileviruses as causal agents of these pathologies (Kitajima et al., 2003; Antonioli-Luizon, 2010), but also indicate that these viruses belong to the same tentative cilevirus species, previously named as PfGSV (Kitajima et al., 1997). In addition, we detected mixed infections between PfGSV and the potyviruses SMV and CABMV in two out of the three samples analyzed by HTS. Accordingly, in the current discussion relevant aspects involving PfGSV-caused diseases emanates from the evaluation of the symptoms showed by the sample Snp1, in which PfGSV was the only virus detected in the HTS analysis, and by other passion fruit plants experimentally infected by PFGSV using viruliferous brevipalpus mites under controlled conditions in a greenhouse (*unpublished results*).

Both the shape and size of detected virus particles, as well as the cytopathic effects observed in the cells of the diseased passion fruit tissues, matched with those previously noted in PfSD-affected plants (Kitajima et al., 2003) and, in general, with those described for cileviruses, and the anatomy of the cells affected by their infections (Freitas-Astúa et al., 2018). PfGSV genomes encompass the six typical cilevirus ORFs subdivided into two RNA molecules with ∼9 and 5 kb (Figure 3) (Freitas-Astúa et al., 2018). Besides the two genomic RNA molecules, four sgRNA molecules were detected in passion fruit infected plants (Figure 5), whereas, a fifth sgRNA, from which P13 protein might be translated, still needs more in wet analyses to confirm its identity. Despite this, the array and number of PfGSV-specific sgRNAs, particularly those derived from its RNA2, resemble more the sgRNA expression pattern revealed by CiLV-C (Pascon et al., 2006), than that shown by CiLV-C2, whose RNA2 sgRNA profile comprises only two molecules (Roy et al., 2013). Moreover, comparisons of nucleotide and deduced amino acid sequences from PfGSV with the cognates from CiLV-C and CiLV-C2 (Table 1) validated that PfGSV isolates are distinct from known viruses and, taken together, support their definitive assignment to a new species of the genus *Cilevirus*, family *Kitaviridae*.

RNA2 of PfGSV isolates Snp1, Snp2, NFo1, and BSB1 harbors a seventh ORF (*p13*) (Figure 3) potentially encoding a protein lacking recognizable homologs. P13 displays 10 residues of Arg, intermingled with other amino acids, rendering a positively charged protein that might account for its likely interaction with either proteins or nucleic acids (Chandana and Venkatesh, 2016). P13 also has Cys residues that make it resemble small cysteine-rich proteins (CRPs) encoded by hordei-, furo-, tobra-, beny-, peclu-, and carlaviruses (Koonin et al., 1991; Yelina et al., 2002). However, unlike those CRP encoded in the 3′-termini of viral genomes, ORF*p13* is placed in the 5′ half of the RNA2 molecule probably suggesting its different transcriptional regulation and accumulation pattern. In-depth bioinformatic analyses of P13 protein indicated it harbors motifs showing the best, although weak, identities with those in proteins involved in transcriptional regulation, e.g., FDB and PGC7/Stella/Dppa3 -like domains (Figure 4). Remarkably, besides P13, P15 also shows Cys residues, which likely contribute to a putative Zn-finger structure (Ramos-González et al., 2016). Studies with CRP from, e.g., tobacco rattle virus and beet necrotic yellow vein virus revealed that Cys residues are essential for protein stability and/or their activity as RNA silencing suppressors (Chiba et al., 2013; Sun et al., 2013; Fernández-Calvino et al., 2016). While P13 expression from the RNA2 of PfGSV_Snp1 still needs to be proven, a thought-provoking question is what would be the likely selective advantage provided by this gene. Clearly contrasting with CiLV-C and CiLV-C2, PfGSV has been identified naturally infecting more than a dozen species of plants belonging to nine families (*unpublished data*), and their lesions in infected plants are less restricted than those produced by the citrus-infecting cileviruses (Freitas-Astúa et al., 2018; Ramos-González et al., 2018) (Figure 1C). It should be noted that the putative allele of the *p13* ORF in the isolate BJL1 (BJL1 allele) produces a truncated version of the protein with ∼11 kDa. Further variability studies including more than 30 isolates are underway in our laboratory aiming to assess the prevalence of the *p13* alleles in the PfGSV population. Besides its presence in four out of the five isolates included in this study, preliminary results indicate the predominance of the Snp1 type (*unpublished data*).

A broader examination of the first third of the PfGSV RNA2 molecule highlights the variability of this region in PfGSV and in general across cileviruses. Considering PfGSV as a reference, this stretch of nucleotide sequence (*i*) contains two divergent alleles for the *p13* ORF (represented by the alleles Snp1 or BJL1), (*ii*) is 17–43% shorter than those in other cileviruses, (*iii*) includes the *p13* ORF that is absent in CiLV-C and CiLV-C2, and (*iv*) accommodates the *p15* ORF, the less conserved among the cileviruses (Table 1). Variability between the RNA2 IR in CiLV-C and CiLV-C2 has been previously emphasized (Melzer et al., 2013b; Roy et al., 2013; Ramos-González et al., 2016). CiLV-C and CiLV-C2 harbor a putative small ORF encoding a 7 kDa protein whose position inside the IR reveals a contrasting structural array between the IR of these two viruses. Early studies on the IR led us to hypothesize the occurrence of continuous illegitimate (non-homologous) recombination processes inter- or intra-species of cileviruses (Ramos-González et al., 2016).

Phylogenetic inferences using RdRp confirmed previous reports indicating close genetic and evolutionary links between plant-infecting viruses of the family *Kitaviridae* and several arthropod-infecting viruses (Figure 6) (Vasilakis et al., 2013; Kallies et al., 2014; Nunes et al., 2017; Kondo et al., 2019; Vinokurov and Koloniuk, 2019). Particularly, the analysis of the RdRp tree indicated that kitavirids might have emerged via a divergent lineage from which other plant-infected viruses, e.g., members of the family *Virgaviridae*, have likely evolved, further underpinning that the diversity of arthropod viruses is the source from where several plants and animal viruses evolved (Shi et al., 2016; Dolja and Koonin, 2018). Through combined analyses based on the RdRp tree and the genome structure of known negeviruses, negevirus-like viruses, and members of the family *Kitaviridae*, it might be speculated that their putative direct ancestors colonized arthropods and displayed unsegmented ss(+)RNA genome with a poly-A tail and three major ORFs arranged in the following order: 5′-ORF1 (RdRp)-ORF2 (putative glycoprotein)-ORF3 (SP24 motif-containing protein)-3′. While circulating negeviruses and negevirus-like viruses keep this general genome structure (Vasilakis et al., 2013), kitavirids show segmented genomes where the orthologs of ORF1 and ORFs 2–3 lie in distinct RNA molecules. In particular, cileviruses might epitomize one of the outgrowths of this evolutionary process in which the high-variable 5′-end of the RNA2, placed upstream the *p61* ORF (putative glycoprotein), represents a block of sequence that lacks in the genome of the known negeviruses and negevirus-like viruses.

The shaping of current cilevirus genomes might have undergone the action of evolutionary forces biased not only by their plant hosts but also by their vectors. Horizontal gene transfer among viruses, or even between viruses and plants, has been suggested as a suitable mechanism for the acquisitions of new genes by plant virus genomes, as properly explained in the case of the binary movement block genes of the kitavirus hibiscus green spot virus 2 (Solovyev and Morozov, 2017). Moreover, blunerviruses and cileviruses encode a 30K MP displaying their best identities with those from virgavirids (Supplementary Figure S4) from which it could likely have been integrated by recombination (Quito-Avila et al., 2013). These two examples suggest that gene acquisition processes could have happened before the diversification of cileviruses and blunerviruses or, otherwise, they came about more than twice during the evolution of current kitavirids. This latter scenario seems to fit better with the speciation processes from two different nodes suggested by the RdRp-based analyses (Figures 6, 7).

Moreover, brevipalpus mites, the known vectors of cileviruses, also act as hosts and vectors of viruses grouped in the genus *Dichorhavirus*, one of the two genera of rhabdoviruses showing segmented genomes (Dietzgen et al., 2018). Genome segmentation of an ancestral mononegavirus into dichorhaviruses has been suggested as likely linked to their unique vector (Kondo et al., 2017). If any, monopartite nege-like viruses infecting brevipalpus mites have not been detected yet. However, besides a number of negeviruses (Agboli et al., 2019), Saiwaicho virus (Medd et al., 2018), aphis glycine virus 3 (Feng et al., 2017), and *Tetranychus urticae* kitavirus (Niu et al., 2019), have been recovered from herbivore arthropods. In such a way, the role of arthropods acting as a bridge between ancestral nege-like viruses and plants seems to be probable.

In this work, we have revealed the genomic sequence of viruses belonging to a putative new species of cilevirus. Deeper studies on molecular variability and prevalence of PfGSV isolates, degree of susceptibility of passion fruit cultivars to virus infection and vector infestation, specificity of the virus-mite interactions, relationship between dynamic of mite populations, e.g., density and seasonal fluctuations, and plant symptom severity will provide us with robust tools to detect and control the infection by PfGSV and to reveal the factors that determine the outbreak of two different diseases with the same etiological agent. Our current study has expanded our knowledge about cileviruses since PfGSV is only the third virus of the genus with a complete genome revealed and, so far, the only one able to infect passion fruit plants. This latter fact is of particular significance because differently from CiLV-C and CiLV-C2, whose primary host is sweet orange (*Citrus sinensis*) trees introduced in America only after the colonization ca. 500 years ago, the mean host of PfGSV is native to South America (Ocampo Pérez and Coppens d’Eeckenbrugge, 2017). If, as it is speculated, South America is the center of origin of brevipalpus-transmitted viruses (Freitas-Astúa et al., 2018), a group that besides the cileviruses includes the ss(−)RNA bipartite viruses of the genus *Dichorhavirus* (Dietzgen et al., 2018), virus-host co-evolutionary history of PfGSV is much larger than that of the CiLV-C or CiLV-C2. More importantly, they might shed light on the complex evolutionary processes involving kitavirids and related arthropod-infecting viruses. Comprehensively, high variability involving the presence of orphan or taxonomically restricted genes in the RNA2 of cileviruses launch these viruses as an excellent system to study the mechanisms determining the birth of new genes, as well as, the role of these genes enabling the viral fitness to novel conditions (Khalturin et al., 2009; Schlötterer, 2015; Johnson, 2018; Van Oss and Carvunis, 2019).

## Data Availability Statement

The datasets generated for this study can be found in the PfGSV_Snp1 RNA1: MK804171 and RNA2: MK804172, PfGSV_BSB1 RNA1: MK804173 and RNA2: MK804174, PfGSV_BJL1 RNA1: MK804175 and RNA2: MK804176, PfGSV_Snp2 RNA1: MN746810 and RNA2: MN746811, PfGSV_NFo1 RNA1: MN746812 and RNA2: MN746813, SMV_BSB1: MN124783, and CABMV_BJL1: MN124782.

## Author Contributions

PR-G conceptualized and wrote the original draft. PR-G and JF-A worked on the formal analysis, supervised the study, wrote reviewed and edited the manuscript. JF-A and EK were responsible for funding acquisition. PR-G, CC-J, EK, and GS worked on the investigation and methodology. JF-A, RH, and EK were responsible for the resources.

## Conflict of Interest

The authors declare that the research was conducted in the absence of any commercial or financial relationships that could be construed as a potential conflict of interest.

## Footnotes

1 https://talk.ictvonline.org/taxonomy/

2 ftp://ftp.ncbi.nih.gov/genomes/Viruses/all.fna.tar.gz

3 https://www.ncbi.nlm.nih.gov/orffinder/

4 https://www.ncbi.nlm.nih.gov/sparkle

5 https://pfam.xfam.org/

6 http://www.cbs.dtu.dk/services/TMHMM/

7 https://web.expasy.org/compute_pi/

8 https://toolkit.tuebingen.mpg.de
