# Supplementary material for: Passion Fruit Green Spot Virus Genome Harbors a New Orphan ORF and Highlights the Flexibility of the 5′-End of the RNA2 Segment Across Cileviruses
Source: Front Microbiol. 2020 Feb 14;11:206. doi: 10.3389/fmicb.2020.00206 (PMC7033587; doi:10.3389/fmicb.2020.00206)
Supplement: Supplementary file 1 [file Image_1.pdf]

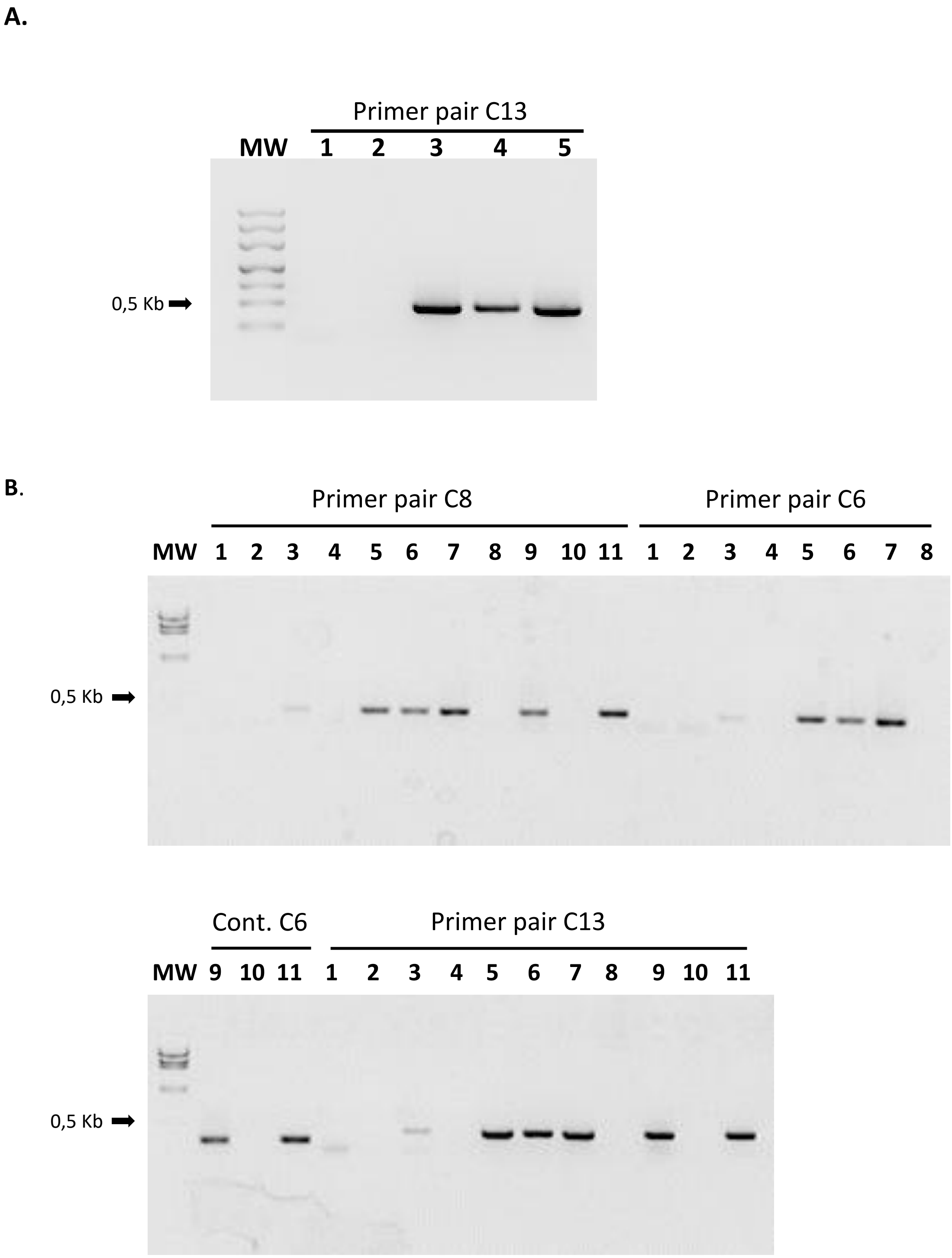

Supplementary Figure 1. **Passion fruit green spot virus detection by RT-PCR**. 1% agarose gel electrophoresis of RT-PCR products. **(A)** Detection of PfGSV in symptomatic passion fruit plants collected in Sinop, Brasilia and Bom Jesus da Lapa, Brazil. MW: Molecular weight marker, M1121 Ladder (Sinapse Biotechnology, Brazil); Lane 1: Reverse-transcription blank; 2: PCR blank; 3: *Passiflora* sp. isolate BJL1; and 4: *Passiflora* sp. isolate BSB1; 5: *Passiflora* sp. from Sinop, MT. PCR assays were carried out using the C13 primer pair. **(B)** Detection of PfGSV in symptomatic passion fruit plants collected in Sinop, Brasilia and Bom Jesus da Lapa, Brazil. MW: Molecular weight marker, Lambda/Hind III (Thermo Fisher Scientific); Lane 1: Reverse-transcription blank; 2: PCR blank; Lines 3 & 4, 5 & 6 and 7 & 8: leaf and stem samples, respectively, of plants 1, 2 and 3. PCR primer pairs were as follow: C8, specific for MP ORF (expected amplicon 245 bp); C6,

specific for p24 ORF (expected amplicon 299 bp); and C13, specific for RdRp ORF (expected amplicon 443 bp).
