# Supplementary material for: Passion Fruit Green Spot Virus Genome Harbors a New Orphan ORF and Highlights the Flexibility of the 5′-End of the RNA2 Segment Across Cileviruses
Source: Front Microbiol. 2020 Feb 14;11:206. doi: 10.3389/fmicb.2020.00206 (PMC7033587; doi:10.3389/fmicb.2020.00206)
Supplement: Supplementary file 2 [file Image_2.pdf]

A.

The conservation scoring is performed by PRALINE. The scoring scheme works from 0 for the least conserved alignment position, up to 10 for the most conserved alignment position. The colour assignments are:

Unconserved 0 1 2 3 4 5 6 7 8 9 10 **Conserved**

```
            . . . . . . . . . 10 . . . . . . . . . 20 . . . . . . . . . 30 . . . . . . . . . 40 . . . . . . . . . 50
CiLV-C_CRD  -MSIVTFTLT DPSSALIAEI MQAIERHNVS --VPEGLRDI SKPTKKKQQS
CiLV-C_SJP  MS-IVTFTLT DPSSALIAEI MQAKERHNVS VPEGLR--NF SKPDKKKQQS
CiLV-C2_Co  MSNIVSFSLS NPSPALIAEI MDAISRHGLN VPAGLAQAPV QRQRQVRQPQ
CiLV-C2_Hw  MSNVVSFSLS NPSPALIAEI MDAISRHGLN VPAGLIQAPA QRPRQVRQPQ
PfGSV_Snp   MSNIVTFSLS NPSPALISEI MDAISRHGMD VPAGLARVPN QRARPVRQQQ
PfGSV_BJL   MSNIVTFSLS NPSPALISEI MDAISRHGMD VPAGLARVPN QRARPVRQQQ
PfGSV_BSB   MSNIVTFSLS NPSPALISEI MDAISRHGMD VPAGLARVPN QRVRPVRQQQ
Consistency 7869*8*8*8 7**6***7** *7*77**776 7767754363 78465 68*67

            . . . . . . . . . 60 . . . . . . . . . 70 . . . . . . . . . 80 . . . . . . . . . 90 . . . . . . . . . 100
CiLV-C_CRD  QPQQLSRASA RPQQLQPGPS GYQAKKPAKQ KAEVVK--PK QKQLAPPINK
CiLV-C_SJP  QPQQPSRASA RSQQLQPSTS GYQAKKPGKQ KVEVVPQKLK QKQPAAPINK
CiLV-C2_Co  IPPPQPRQAP RQRVNPP--- ----ARAAPQ QAQNRP--AV PPQIGLPIRR
CiLV-C2_Hw  IPPNQPRQAP RQRVNPP--- ----ARPTPQ QAQNYP--AA PPQIGLPIRR
PfGSV_Snp   QPPQ-QRQAP RQRVDPP--- -VRAERAAPP QRVVQP---- ---PRGLAVR
PfGSV_BJL   QPPQ-QRQAP RQRVDPP--- -VRAERAAPP QRVVQP---- ---PRGLAVR
PfGSV_BSB   PPPQ-QRQAP RQRVDPP--- -VRAERAAPP QRVVQP---- ---PRGLAVR
Consistency 4*6615*686 *67646*000 0234586766 7545480011 1124434638

            . . . . . . . . . 110. . . . . . . . . 120. . . . . . . . . 130. . . . . . . . . 140. . . . . . . . . 150
CiLV-C_CRD  KAAKAKLYGL EQHCPKYAEA KGLQKQIGMT YYKISEPYAL PDFKVMEASE
CiLV-C_SJP  KAAKAKLYGL EQHCPKYAEA KGLQKQIGMT YYKISEPYAL PDFKVMEASE
CiLV-C2_Co  VAVAGGSVGS VEHQEYYRSL PGYSKTYGCT KYNPNTPYTI VGFKLSEPSE
CiLV-C2_Hw  VAVAGNSVGS VEHQEYYRSL PGYSKTYGCT KYNPNTPYTI VGFKLSEPSE
PfGSV_Snp   EAPAGDVVVP LEHQEYYRSL PGFARSYGCT KYNPLNPYTI TGFRLTDPAD
PfGSV_BJL   EAPAGDVVVP LEHQEYYRSL PGFARSYGCT KYNPLNPYTI TGFRLTDPAD
PfGSV_BSB   EAPAGDVVVP LEHQEYYRSL PGFARSYGCT KYNPLNPYTI TGFRLTDPAD
Consistency 5*46745644 48*566*677 6*66866*6* 6*7545**78 46*8857677

            . . . . . . . . . 160. . . . . . . . . 170. . . . . . . . . 180. . . . . . . . . 190. . . . . . . . . 200
CiLV-C_CRD  DLVAVSEKDP MGSFEKRLYS MGFPKRPIKN VVPVFEFSDH YIVVFFPGSN
CiLV-C_SJP  DLVAVNEKDP MGTFEKRLYS MGFPKRPIKN VIPVFEFSDH YIVVFFPGSN
CiLV-C2_Co  ELAVVDAKDL KASFKRRLKS LGFPSCGTDS IIVAHEYPDH YIAVIFPGAP
CiLV-C2_Hw  ELAVVDAKDL KASFKRRLKS LGFPTCGTDS IIVAHEYPDH YIAVIFPGAP
PfGSV_Snp   ELVEVDPTAL RSSFKQRLAS LGFPTCNIES VVVAHEYPDH YFVVLFPGAP
PfGSV_BJL   ELVEVDPTAL RSSFKQRLAS LGFPTCNIES VVVAHEYPDH YFVVLFPGAP
PfGSV_BSB   ELVEVDPTAL RSSFKQRLAS LGFPTCNIES VVVAHEYPDH YFVVLFPGAP
Consistency 8*75*74655 568*76**4* 8***654667 99676*86** *67*6***86

            . . . . . . . . . 210. . . . . . . . . 220. . . . . . . . . 230. . . . . . . . . 240. . . . . . . . . 250
CiLV-C_CRD  AEIVKNVPKD SVSDYAEAQL AALLAARQQI NQIHELGDIL PTNYLNVLDS
CiLV-C_SJP  AEIVKNIPKD SVADYAEAQL AALLAARQQI NQIHDLGDIL PTNYLNVLDS
CiLV-C2_Co  YQLPVECPKD KVASPEDAKK VALAGCIRDI NSVTDVRGIL PVSYLELERL
CiLV-C2_Hw  YQLPVECPKD KVTSPEDAKK VALAGCIRDI NSVTDVRGIL PISYLELEKL
PfGSV_Snp   YEMPVNCPKD RVRSPKDAKD IALAGCLHDI NRITDVRGIL PYNYLRLKNL
PfGSV_BJL   YEMPVNCPKD RVRSPKDAKD IALAGCLHDI NRITDVRGIL PYNYLRLKNL
PfGSV_BSB   YEMPVNCPKD RVRSPKDAKD IALAGCLHDI NRITDVRGIL PYNYLRLKNL
Consistency 6876676*** 6*57558*73 6**777557* *5968866** *47**58456

            . . . . . . . . . 260. . . . . . . . . 270. . .
CiLV-C_CRD  GTQDVVVSDE EDDSDSAQ-- ---
CiLV-C_SJP  GTQD--VVVS DGDDESQSEQ ---
CiLV-C2_Co  GTPPPLVLLP DDDDDQQVDE QEE
CiLV-C2_Hw  GMPPPLVLLP DEDDQQAEEQ GE-
PfGSV_Snp   GTPPPLELAP LDEQVEGGDE ---
PfGSV_BJL   GTPPPLELAP LDEQVEGGDE ---
PfGSV_BSB   GTPPPLELAP LDEQVEGGDE ---
Consistency *866565646 3775464355 000
```

B.

The colour assignments have been adapted from the defaults in CLUSTALX (Thompson *et al*, 1997) Abstract :

G, P, S, T	H, K, R	F, W, Y	I, L, M, V

```
           .........10.........20.........30.........40.........50
CiLV-C_CRD -MSIVTFTLT DPSSALIAEI MQAIERHNVS --VPEGLRDI SKPTKKKQQS
CiLV-C_SJP MS-IVTFTLT DPSSALIAEI MQAKERHNVS VPEGLR--NF SKPDKKKQQS
CiLV-C2_Co MSNIVSFSLS NPSPALIAEI MDAISRHGLN VPAGLAQAPV QRQRQVRQPQ
CiLV-C2_Hw MSNVVSFSLS NPSPALIAEI MDAISRHGLN VPAGLIQAPA QRPRQVRQPQ
PfGSV_Snp  MSNIVTFSLS NPSPALISEI MDAISRHGMD VPAGLARVPN QRARPVRQQQ
PfGSV_BJL  MSNIVTFSLS NPSPALISEI MDAISRHGMD VPAGLARVPN QRARPVRQQQ
PfGSV_BSB  MSNIVTFSLS NPSPALISEI MDAISRHGMD VPAGLARVPN QRVRPVRQQQ

           .........60.........70.........80.........90.........100
CiLV-C_CRD QPQQLSRASA RPQQLQPGPS GYQAKKPAKQ KAEVVK--PK QKQLAPPINK
CiLV-C_SJP QPQQPSRASA RSQQLQPSTS GYQAKKPGKQ KVEVVPQKLK QKQPAAPINK
CiLV-C2_Co IPPPQPRQAP RQRVNPP--- ----ARAAPQ QAQNRP--AV PPQIGLPIRR
CiLV-C2_Hw IPPNQPRQAP RQRVNPP--- ----ARPTPQ QAQNYP--AA PPQIGLPIRR
PfGSV_Snp  QPPQ-QRQAP RQRVDPP--- -VRAERAAPP QRVVQP---- ---PRGLAVR
PfGSV_BJL  QPPQ-QRQAP RQRVDPP--- -VRAERAAPP QRVVQP---- ---PRGLAVR
PfGSV_BSB  PPPQ-QRQAP RQRVDPP--- -VRAERAAPP QRVVQP---- ---PRGLAVR

           .........110.........120.........130.........140.........150
CiLV-C_CRD KAAKAKLYGL EQHCPKYAEA KGLQKQIGMT YYKISEPYAL PDFKVMEASE
CiLV-C_SJP KAAKAKLYGL EQHCPKYAEA KGLQKQIGMT YYKISEPYAL PDFKVMEASE
CiLV-C2_Co VAVAGGSVGS VEHQEYYRSL PGYSKTYGCT KYNPNTPYTI VGFKLSEPSE
CiLV-C2_Hw VAVAGNSVGS VEHQEYYRSL PGYSKTYGCT KYNPNTPYTI VGFKLSEPSE
PfGSV_Snp  EAPAGDVVVP LEHQEYYRSL PGFARSYGCT KYNPLNPYTI TGFRLTDPAD
PfGSV_BJL  EAPAGDVVVP LEHQEYYRSL PGFARSYGCT KYNPLNPYTI TGFRLTDPAD
PfGSV_BSB  EAPAGDVVVP LEHQEYYRSL PGFARSYGCT KYNPLNPYTI TGFRLTDPAD

           .........160.........170.........180.........190.........200
CiLV-C_CRD DLVAVSEKDP MGSFEKRLYS MGFPKRPIKN VVPVFEFSDH YIVVFFPGSN
CiLV-C_SJP DLVAVNEKDP MGTFEKRLYS MGFPKRPIKN VIPVFEFSDH YIVVFFPGSN
CiLV-C2_Co ELAVVDAKDL KASFKRRLKS LGFPSCGTDS IIVAHEYPDH YIAVIFPGAP
CiLV-C2_Hw ELAVVDAKDL KASFKRRLKS LGFPTCGTDS IIVAHEYPDH YIAVIFPGAP
PfGSV_Snp  ELVEVDPTAL RSSFKQRLAS LGFPTCNIES VVVAHEYPDH YFVVLFPGAP
PfGSV_BJL  ELVEVDPTAL RSSFKQRLAS LGFPTCNIES VVVAHEYPDH YFVVLFPGAP
PfGSV_BSB  ELVEVDPTAL RSSFKQRLAS LGFPTCNIES VVVAHEYPDH YFVVLFPGAP

           .........210.........220.........230.........240.........250
CiLV-C_CRD AEIVKNVPKD SVSDYAEAQL AALLAARQQI NQIHELGDIL PTNYLNVLDS
CiLV-C_SJP AEIVKNIPKD SVADYAEAQL AALLAARQQI NQIHDLGDIL PTNYLNVLDS
CiLV-C2_Co YQLPVECPKD KVASPEDAKK VALAGCIRDI NSVTDVRGIL PVSYLELERL
CiLV-C2_Hw YQLPVECPKD KVTSPEDAKK VALAGCIRDI NSVTDVRGIL PISYLELEKL
PfGSV_Snp  YEMPVNCPKD RVRSPKDAKD IALAGCLHDI NRITDVRGIL PYNYLRLKNL
PfGSV_BJL  YEMPVNCPKD RVRSPKDAKD IALAGCLHDI NRITDVRGIL PYNYLRLKNL
PfGSV_BSB  YEMPVNCPKD RVRSPKDAKD IALAGCLHDI NRITDVRGIL PYNYLRLKNL

           .........260.........270...
CiLV-C_CRD GTQDVVVSDE EDDSDSAQ-- ---
CiLV-C_SJP GTQD--VVVS DGDDESQSEQ ---
CiLV-C2_Co GTPPPLVLLP DDDDDQQVDE QEE
CiLV-C2_Hw GMPPPLVLLP DEDDQQAEEQ GE-
PfGSV_Snp  GTPPPLELAP LDEQVEGGDE ---
PfGSV_BJL  GTPPPLELAP LDEQVEGGDE ---
PfGSV_BSB  GTPPPLELAP LDEQVEGGDE ---
```

C.

The 3-state (H, E C) secondary structure for each sequence is represented by a colour. If a sequence in the alignment has no colours assigned, this means that either there is no DSSP information available (if this was requested), or that no prediction was possible for that sequence (if this was requested).
The colour assignments are:

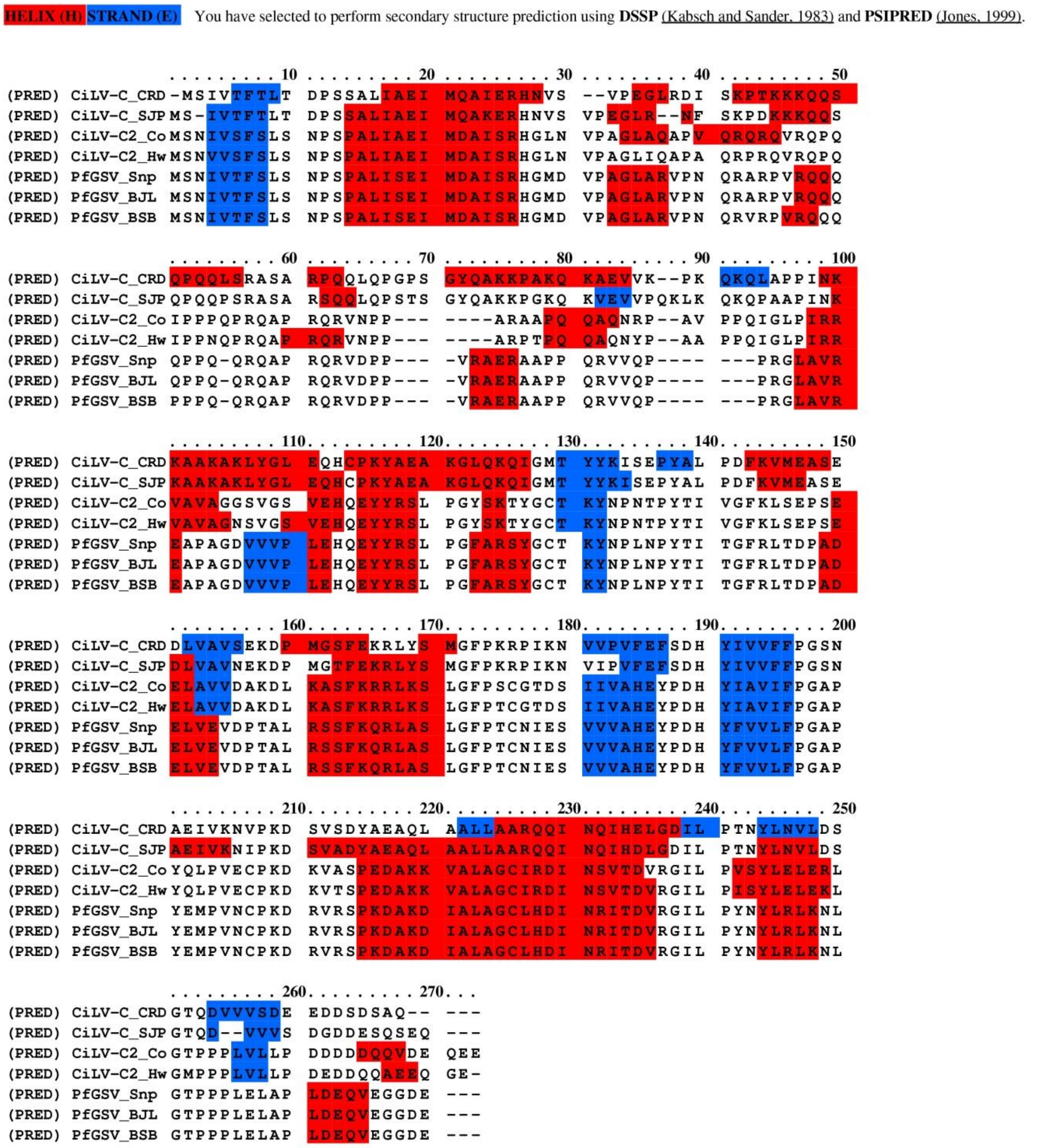

Supplementary Figure 2. **Alignment of P29 proteins of cileviruses**. PRALINE software results indicate **(A)** the residue conservation scoring, **(B)** 3-state (H: Helix, E: Strand, C: Coil) secondary structure, and **(C)** and type of residues.
