# Supplementary material for: Passion Fruit Green Spot Virus Genome Harbors a New Orphan ORF and Highlights the Flexibility of the 5′-End of the RNA2 Segment Across Cileviruses
Source: Front Microbiol. 2020 Feb 14;11:206. doi: 10.3389/fmicb.2020.00206 (PMC7033587; doi:10.3389/fmicb.2020.00206)
Supplement: Supplementary file 3 [file Image_3.pdf]

A.

The conservation scoring is performed by PRALINE. The scoring scheme works from 0 for the least conserved alignment position, up to 10 for the most conserved alignment position.
The colour assignments are:

Unconserved 0 1 2 3 4 5 6 7 8 9 10 **Conserved**

```
            . . . . . . . . . 10 . . . . . . . . . 20 . . . . . . . . . 30 . . . . . . . . . 40 . . . . . . . . . 50
CiLV-C_CRD  MDAQLLQANK RLLRRAANVR QRYKMLATES FVADIKQILL RFIQKPNVII
CiLV-C_SJP  MDAQLLQTNK RLLRRAANVR QKYKVLASDS FVADIKQILL RFIPKPNVII
PfGSV_Snp   MDPRFLRG-R SLVTNVTDKR ERLKSKASFS LLSDIQQILL RYIQKPYVLL
PfGSV_BJL   MDPRFLRG-R SLVTNVTDKR ERLKSKASFS LLSDIQQILL RYIQKPYVLL
PfGSV_BSB   MDPRFLRG-R SLVTNVTDKR ERLKSKASFS LLSDIQQILL RYIQKPYVLL
CiLV-C2_Co  MDQRFLRG-- ASVLNLSNKR EKLKTNASVS LLSDIQQILL RYIQKPYVLL
CiLV-C2_Hw  MDQRFLRG-- ASVLNLSNKR DKLKTNASVS LLSDIQQILL RYIQKPYVLL
CiLV-C2_Fla MDQRFLRG-- ASVLNLSNKR DKLKTNASVS LLSDIQQILL RYIQKPYVLL
Consistency **487*8602 658476676* 787*54*84* 788**8**** *8*8**6*88

            . . . . . . . . . 60 . . . . . . . . . 70 . . . . . . . . . 80 . . . . . . . . . 90 . . . . . . . . . 100
CiLV-C_CRD  MYISVLVLFA AHIDSNTHDI LDDLAAQFPN NTFIEWAKSN FFRICGALVF
CiLV-C_SJP  MYISVLVLFA AHIDSNTHDI LDDLAAQFPN NTFIEWAKNN FFRICGALVF
PfGSV_Snp   MYACVLVLFA MHIDAGTHDI LDDLAQQFPN NPVIEWARGN FFRLCGALVF
PfGSV_BJL   MYACVLVLFA MHIDAGTHDI LDDLAQQFPN NPVIEWARGN FFRLCGALVF
PfGSV_BSB   MYACVLVLFA MHIDAGTHDI LDDLAQQFPN NPVIEWARGN FFRLCGALVF
CiLV-C2_Co  MYACVLVLFA MHIDAGTHDI LDDLAQQFPN NPVIEWARNN FFRLCGALVF
CiLV-C2_Hw  MYACVLVLFA MHIDAGTHDI LDDLAQQFPN NPVIEWARSN FFRLCGALVF
CiLV-C2_Fla MYACVLVLFA MHIDAGTHDI LDDLAQQFPN NPVIEWARSN FFRLCGALVF
Consistency **76****** 7***87**** *****7**** *77****85* ***8******

            . . . . . . . . . 110. . . . . . . . . 120. . . . . . . . . 130. . . . . . . . . 140. . . . . . . . . 150
CiLV-C_CRD  IPVIIDTEEK HRNYLALVIF VFLMGFPQRS IMEYFIYSIS FHVYAKAKHP
CiLV-C_SJP  IPVIIDTEEK HRNYLALVIF VFLMGFPQRS IMEYFIYSIS FHVYAKAKHP
PfGSV_Snp   IPVITDAQKE HQLYFGMVIG LFLLGFPQRS IFEYFVYSLS LHVYAKSKHP
PfGSV_BJL   IPVITDAQKE HQLYFGMVIG LFLLGFPQRS IFEYFVYSLS LHVYAKSKHP
PfGSV_BSB   IPVITDAQKE HQLYFGMVIG LFLLGFPQRS IFEYFVYSLS LHVYAKSKHP
CiLV-C2_Co  IPVITDARKE HQLYFGMVIA LFLLGFPQRS IFEYFVYSLS LHVYAKSKHP
CiLV-C2_Hw  IPVITDARKE HQLYFGMVIA LFLLGFPQRS IFEYFVYSLS LHVYAKSKHP
CiLV-C2_Fla IPVITDARKE HQSYFGMVIA LFLLGFPQRS IFEYFVYSLS LHVCAKSKHP
Consistency ****7*7688 *85*778**4 8**8****** *7***9**8* 7**7**8***

            . . . . . . . . . 160. . . . . . . . . 170. . . . . . . . . 180. . . . . . . . . 190. . . . . . . . . 200
CiLV-C_CRD  VTRIFIIGAA VFSCVMFGIF TNEQLRKLYA ELPKVPTHPV AVNRVEKVAN
CiLV-C_SJP  VTRIFIVGAA IFSCVMFGIF TNEQLRKLYA ELPKVPTHPV T-NKVEKVAS
PfGSV_Snp   MTRIFILVVA VTSCVLFGVF TNEQLKKLYQ ELPKVPTHPV --NKVEKVVN
PfGSV_BJL   MTRIFILVVA VTSCVLFGVF TNEQLKKLYQ ELPKVPTHPV --NKVEKVVN
PfGSV_BSB   MTRIFILVVA VTSCVLFGVF TNEQLKKLYQ ELPKVPTHPV --NKVEKVVN
CiLV-C2_Co  FTRIFILVIT VVSCVLFGVF TNDQLRKLYQ ELPKVPTHPV --NKVERVVN
CiLV-C2_Hw  FTRVFILVIT VVSCVLFGVF TNDQLRKLYQ ELPKVPTHPV --NRVERVVN
CiLV-C2_Fla FTRVFILVIT VVSCVLFGVF TNDQLRKLYQ ELPKVPTHPV --NRVERVVN
Consistency 5**9**8677 95***8**9* **8**8***7 ********** 00*8**8*78

            . . . . . . . . . 210. . . . .
CiLV-C_CRD  RASRVSTEGT VNFG-
CiLV-C_SJP  RASRVSTEGT INFG-
PfGSV_Snp   RVG-----GQ QQFQG
PfGSV_BJL   RVG-----GQ QQFQG
PfGSV_BSB   RVG-----GQ QQFQG
CiLV-C2_Co  RVG-----GQ QSFSG
CiLV-C2_Hw  RVG-----GQ QPYTG
CiLV-C2_Fla RVG-----GQ QPYTG
Consistency *7700000*7 64845
```

B.

The colour assignments have been adapted from the defaults in CLUSTALX (Thompson *et al*, 1997) Abstract :

G, P, S, T	H, K, R	F, W, Y	I, L, M, V

```
            .........10.........20.........30.........40.........50
CiLV-C_CRD  MDAQLLQANK RLLRRAANVR QRYKMLATES FVADIKQILL RFIQKPNVII
CiLV-C_SJP  MDAQLLQTNK RLLRRAANVR QKYKVLASDS FVADIKQILL RFIPKPNVII
PfGSV_Snp   MDPRFLRG-R SLVTNVTDKR ERLKSKASFS LLSDIQQILL RYIQKPYVLL
PfGSV_BJL   MDPRFLRG-R SLVTNVTDKR ERLKSKASFS LLSDIQQILL RYIQKPYVLL
PfGSV_BSB   MDPRFLRG-R SLVTNVTDKR ERLKSKASFS LLSDIQQILL RYIQKPYVLL
CiLV-C2_Co  MDQRFLRG-- ASVLNLSNKR EKLKTNASVS LLSDIQQILL RYIQKPYVLL
CiLV-C2_Hw  MDQRFLRG-- ASVLNLSNKR DKLKTNASVS LLSDIQQILL RYIQKPYVLL
CiLV-C2_FlaMDQRFLRG-- ASVLNLSNKR DKLKTNASVS LLSDIQQILL RYIQKPYVLL

            .........60.........70.........80.........90.........100
CiLV-C_CRD  MYISVLVLFA AHIDSNTHDI LDDLAAQFPN NTFIEWAKSN FFRICGALVF
CiLV-C_SJP  MYISVLVLFA AHIDSNTHDI LDDLAAQFPN NTFIEWAKNN FFRICGALVF
PfGSV_Snp   MYACVLVLFA MHIDAGTHDI LDDLAQQFPN NPVIEWARGN FFRLCGALVF
PfGSV_BJL   MYACVLVLFA MHIDAGTHDI LDDLAQQFPN NPVIEWARGN FFRLCGALVF
PfGSV_BSB   MYACVLVLFA MHIDAGTHDI LDDLAQQFPN NPVIEWARGN FFRLCGALVF
CiLV-C2_Co  MYACVLVLFA MHIDAGTHDI LDDLAQQFPN NPVIEWARNN FFRLCGALVF
CiLV-C2_Hw  MYACVLVLFA MHIDAGTHDI LDDLAQQFPN NPVIEWARSN FFRLCGALVF
CiLV-C2_FlaMYACVLVLFA MHIDAGTHDI LDDLAQQFPN NPVIEWARSN FFRLCGALVF

            .........110.........120.........130.........140.........150
CiLV-C_CRD  IPVIIDTEEK HRNYLALVIF VFLMGFPQRS IMEYFIYSIS FHVYAKAKHP
CiLV-C_SJP  IPVIIDTEEK HRNYLALVIF VFLMGFPQRS IMEYFIYSIS FHVYAKAKHP
PfGSV_Snp   IPVITDAQKE HQLYFGMVIG LFLLGFPQRS IFEYFVYSLS LHVYAKSKHP
PfGSV_BJL   IPVITDAQKE HQLYFGMVIG LFLLGFPQRS IFEYFVYSLS LHVYAKSKHP
PfGSV_BSB   IPVITDAQKE HQLYFGMVIG LFLLGFPQRS IFEYFVYSLS LHVYAKSKHP
CiLV-C2_Co  IPVITDARKE HQLYFGMVIA LFLLGFPQRS IFEYFVYSLS LHVYAKSKHP
CiLV-C2_Hw  IPVITDARKE HQLYFGMVIA LFLLGFPQRS IFEYFVYSLS LHVYAKSKHP
CiLV-C2_FlaIPVITDARKE HQSYFGMVIA LFLLGFPQRS IFEYFVYSLS LHVCAKSKHP

            .........160.........170.........180.........190.........200
CiLV-C_CRD  VTRIFIIGAA VFSCVMFGIF TNEQLRKLYA ELPKVPTHPV AVNRVEKVAN
CiLV-C_SJP  VTRIFIVGAA IFSCVMFGIF TNEQLRKLYA ELPKVPTHPV T-NKVEKVAS
PfGSV_Snp   MTRIFILVVA VTSCVLFGVF TNEQLKKLYQ ELPKVPTHPV --NKVEKVVN
PfGSV_BJL   MTRIFILVVA VTSCVLFGVF TNEQLKKLYQ ELPKVPTHPV --NKVEKVVN
PfGSV_BSB   MTRIFILVVA VTSCVLFGVF TNEQLKKLYQ ELPKVPTHPV --NKVEKVVN
CiLV-C2_Co  FTRIFILVIT VVSCVLFGVF TNDQLRKLYQ ELPKVPTHPV --NKVERVVN
CiLV-C2_Hw  FTRVFILVIT VVSCVLFGVF TNDQLRKLYQ ELPKVPTHPV --NRVERVVN
CiLV-C2_FlaFTRVFILVIT VVSCVLFGVF TNDQLRKLYQ ELPKVPTHPV --NRVERVVN

            .........210.....
CiLV-C_CRD  RASRVSTEGT VNFG-
CiLV-C_SJP  RASRVSTEGT INFG-
PfGSV_Snp   RVG-----GQ QQFQG
PfGSV_BJL   RVG-----GQ QQFQG
PfGSV_BSB   RVG-----GQ QQFQG
CiLV-C2_Co  RVG-----GQ QSFSG
CiLV-C2_Hw  RVG-----GQ QPYTG
CiLV-C2_FlaRVG-----GQ QPYTG
```

C.

The 3-state (H, E C) secondary structure for each sequence is represented by a colour. If a sequence in the alignment has no colours assigned, this means that either there is no DSSP information available (if this was requested), or that no prediction was possible for that sequence (if this was requested).
The colour assignments are:

HELIX (H) STRAND (E) You have selected to perform secondary structure prediction using **DSSP** (Kabsch and Sander, 1983) and **PSIPRED** (Jones, 1999).

```
                  .........10 .........20 .........30 .........40 .........50
(PRED) CiLV-C_CRD MDAQLLQANK RLLRRAANVR QRYKMLATES FVADIKQILL RFIQKPNVII
(PRED) CiLV-C_SJP MDAQLLQTNK RLLRRAANVR QKYKVLASDS FVADIKQILL RFIPKPNVII
(PRED) PfGSV_Snp  MDPRFLRG-R SLVTNVTDKR ERLKSKASFS LLSDIQQILL RYIQKPYVLL
(PRED) PfGSV_BJL  MDPRFLRG-R SLVTNVTDKR ERLKSKASFS LLSDIQQILL RYIQKPYVLL
(PRED) PfGSV_BSB  MDPRFLRG-R SLVTNVTDKR ERLKSKASFS LLSDIQQILL RYIQKPYVLL
(PRED) CiLV-C2_Co MDQRFLRG-- ASVLNLSNKR EKLKTNASVS LLSDIQQILL RYIQKPYVLL
(PRED) CiLV-C2_Hw MDQRFLRG-- ASVLNLSNKR DKLKTNASVS LLSDIQQILL RYIQKPYVLL
(PRED) CiLV-C2_FlaMDQRFLRG-- ASVLNLSNKR DKLKTNASVS LLSDIQQILL RYIQKPYVLL

                  .........60 .........70 .........80 .........90 .........100
(PRED) CiLV-C_CRD MYISVLVLFA AHIDSNTHDI LDDLAAQFPN NTFIEWAKSN FFRICGALVF
(PRED) CiLV-C_SJP MYISVLVLFA AHIDSNTHDI LDDLAAQFPN NTFIEWAKNN FFRICGALVF
(PRED) PfGSV_Snp  MYACVLVLFA MHIDAGTHDI LDDLAQQFPN NPVIEWARGN FFRLCGALVF
(PRED) PfGSV_BJL  MYACVLVLFA MHIDAGTHDI LDDLAQQFPN NPVIEWARGN FFRLCGALVF
(PRED) PfGSV_BSB  MYACVLVLFA MHIDAGTHDI LDDLAQQFPN NPVIEWARGN FFRLCGALVF
(PRED) CiLV-C2_Co MYACVLVLFA MHIDAGTHDI LDDLAQQFPN NPVIEWARNN FFRLCGALVF
(PRED) CiLV-C2_Hw MYACVLVLFA MHIDAGTHDI LDDLAQQFPN NPVIEWARSN FFRLCGALVF
(PRED) CiLV-C2_FlaMYACVLVLFA MHIDAGTHDI LDDLAQQFPN NPVIEWARSN FFRLCGALVF

                  .........110.........120.........130.........140.........150
(PRED) CiLV-C_CRD IPVIIDTEEK HRNYLALVIF VFLMGFPQRS IMEYFIYSIS FHVYAKAKHP
(PRED) CiLV-C_SJP IPVIIDTEEK HRNYLALVIF VFLMGFPQRS IMEYFIYSIS FHVYAKAKHP
(PRED) PfGSV_Snp  IPVITDAQKE HQLYFGMVIG LFLLGFPQRS IFEYFVYSLS LHVYAKSKHP
(PRED) PfGSV_BJL  IPVITDAQKE HQLYFGMVIG LFLLGFPQRS IFEYFVYSLS LHVYAKSKHP
(PRED) PfGSV_BSB  IPVITDAQKE HQLYFGMVIG LFLLGFPQRS IFEYFVYSLS LHVYAKSKHP
(PRED) CiLV-C2_Co IPVITDARKE HQLYFGMVIA LFLLGFPQRS IFEYFVYSLS LHVYAKSKHP
(PRED) CiLV-C2_Hw IPVITDARKE HQLYFGMVIA LFLLGFPQRS IFEYFVYSLS LHVYAKSKHP
(PRED) CiLV-C2_FlaIPVITDARKE HQSYFGMVIA LFLLGFPQRS IFEYFVYSLS LHVCAKSKHP

                  .........160.........170.........180.........190.........200
(PRED) CiLV-C_CRD VTRIFIIGAA VFSCVMFGIF TNEQLRKLYA ELPKVPTHPV AVNRVEKVAN
(PRED) CiLV-C_SJP VTRIFIVGAA IFSCVMFGIF TNEQLRKLYA ELPKVPTHPV T-NKVEKVAS
(PRED) PfGSV_Snp  MTRIFILVVA VTSCVLFGVF TNEQLKKLYQ ELPKVPTHPV --NKVEKVVN
(PRED) PfGSV_BJL  MTRIFILVVA VTSCVLFGVF TNEQLKKLYQ ELPKVPTHPV --NKVEKVVN
(PRED) PfGSV_BSB  MTRIFILVVA VTSCVLFGVF TNEQLKKLYQ ELPKVPTHPV --NKVEKVVN
(PRED) CiLV-C2_Co FTRIFILVIT VVSCVLFGVF TNDQLRKLYQ ELPKVPTHPV --NKVERVVN
(PRED) CiLV-C2_Hw FTRVFILVIT VVSCVLFGVF TNDQLRKLYQ ELPKVPTHPV --NRVERVVN
(PRED) CiLV-C2_FlaFTRVFILVIT VVSCVLFGVF TNDQLRKLYQ ELPKVPTHPV --NRVERVVN

                  .........210.....
(PRED) CiLV-C_CRD RASRVSTEGT VNFG-
(PRED) CiLV-C_SJP RASRVSTEGT INFG-
(PRED) PfGSV_Snp  RVG-----GQ QQFQG
(PRED) PfGSV_BJL  RVG-----GQ QQFQG
(PRED) PfGSV_BSB  RVG-----GQ QQFQG
(PRED) CiLV-C2_Co RVG-----GQ QSFSG
(PRED) CiLV-C2_Hw RVG-----GQ QPYTG
(PRED) CiLV-C2_FlaRVG-----GQ QPYTG
```

D. P24 protein of cileviruses. Transmembrane structure.

The transmembrane structure for each sequence is represented by a colour. If a sequence in the alignment has no colours assigned, this means that no prediction was possible for that sequence (if this was requested).
The colour assignment is:

TM SEGMENT (T) You have selected to perform transmembrane structure prediction using **PHOBIUS** (Kall et. al, 2005).

```
                          .........10 .........20 .........30 .........40 .........50
(PRED) CiLV-C_CRD   MDAQLLQANK RLLRRAANVR QRYKMLATES FVADIKQILL RFIQKPNVII
(PRED) CiLV-C_SJP   MDAQLLQTNK RLLRRAANVR QKYKVLASDS FVADIKQILL RFIPKPNVII
(PRED) PfGSV_Snp    MDPRFLRG-R SLVTNVTDKR ERLKSKASFS LLSDIQQILL RYIQKPYVLL
(PRED) PfGSV_BJL    MDPRFLRG-R SLVTNVTDKR ERLKSKASFS LLSDIQQILL RYIQKPYVLL
(PRED) PfGSV_BSB    MDPRFLRG-R SLVTNVTDKR ERLKSKASFS LLSDIQQILL RYIQKPYVLL
(PRED) CiLV-C2_Co   MDQRFLRG-- ASVLNLSNKR EKLKTNASVS LLSDIQQILL RYIQKPYVLL
(PRED) CiLV-C2_Hw   MDQRFLRG-- ASVLNLSNKR DKLKTNASVS LLSDIQQILL RYIQKPYVLL
(PRED) CiLV-C2_Fla  MDQRFLRG-- ASVLNLSNKR DKLKTNASVS LLSDIQQILL RYIQKPYVLL

                          .........60 .........70 .........80 .........90 .........100
(PRED) CiLV-C_CRD   MYISVLVLFA AHIDSNTHDI LDDLAAQFPN NTFIEWAKSN FFRICGALVF
(PRED) CiLV-C_SJP   MYISVLVLFA AHIDSNTHDI LDDLAAQFPN NTFIEWAKNN FFRICGALVF
(PRED) PfGSV_Snp    MYACVLVLFA MHIDAGTHDI LDDLAQQFPN NPVIEWARGN FFRLCGALVF
(PRED) PfGSV_BJL    MYACVLVLFA MHIDAGTHDI LDDLAQQFPN NPVIEWARGN FFRLCGALVF
(PRED) PfGSV_BSB    MYACVLVLFA MHIDAGTHDI LDDLAQQFPN NPVIEWARGN FFRLCGALVF
(PRED) CiLV-C2_Co   MYACVLVLFA MHIDAGTHDI LDDLAQQFPN NPVIEWARNN FFRLCGALVF
(PRED) CiLV-C2_Hw   MYACVLVLFA MHIDAGTHDI LDDLAQQFPN NPVIEWARSN FFRLCGALVF
(PRED) CiLV-C2_Fla  MYACVLVLFA MHIDAGTHDI LDDLAQQFPN NPVIEWARSN FFRLCGALVF

                          .........110.........120.........130.........140.........150
(PRED) CiLV-C_CRD   IPVIIDTEEK HRNYLALVIF VFLMGFPQRS IMEYFIYSIS FHVYAKAKHP
(PRED) CiLV-C_SJP   IPVIIDTEEK HRNYLALVIF VFLMGFPQRS IMEYFIYSIS FHVYAKAKHP
(PRED) PfGSV_Snp    IPVITDAQKE HQLYFGMVIG LFLLGFPQRS IFEYFVYSLS LHVYAKSKHP
(PRED) PfGSV_BJL    IPVITDAQKE HQLYFGMVIG LFLLGFPQRS IFEYFVYSLS LHVYAKSKHP
(PRED) PfGSV_BSB    IPVITDAQKE HQLYFGMVIG LFLLGFPQRS IFEYFVYSLS LHVYAKSKHP
(PRED) CiLV-C2_Co   IPVITDARKE HQLYFGMVIA LFLLGFPQRS IFEYFVYSLS LHVYAKSKHP
(PRED) CiLV-C2_Hw   IPVITDARKE HQLYFGMVIA LFLLGFPQRS IFEYFVYSLS LHVYAKSKHP
(PRED) CiLV-C2_Fla  IPVITDARKE HQSYFGMVIA LFLLGFPQRS IFEYFVYSLS LHVCAKSKHP

                          .........160.........170.........180.........190.........200
(PRED) CiLV-C_CRD   VTRIFIIGAA VFSCVMFGIF TNEQLRKLYA ELPKVPTHPV AVNRVEKVAN
(PRED) CiLV-C_SJP   VTRIFIVGAA IFSCVMFGIF TNEQLRKLYA ELPKVPTHPV T-NKVEKVAS
(PRED) PfGSV_Snp    MTRIFILVVA VTSCVLFGVF TNEQLKKLYQ ELPKVPTHPV --NKVEKVVN
(PRED) PfGSV_BJL    MTRIFILVVA VTSCVLFGVF TNEQLKKLYQ ELPKVPTHPV --NKVEKVVN
(PRED) PfGSV_BSB    MTRIFILVVA VTSCVLFGVF TNEQLKKLYQ ELPKVPTHPV --NKVEKVVN
(PRED) CiLV-C2_Co   FTRIFILVIT VVSCVLFGVF TNDQLRKLYQ ELPKVPTHPV --NKVERVVN
(PRED) CiLV-C2_Hw   FTRVFILVIT VVSCVLFGVF TNDQLRKLYQ ELPKVPTHPV --NRVERVVN
(PRED) CiLV-C2_Fla  FTRVFILVIT VVSCVLFGVF TNDQLRKLYQ ELPKVPTHPV --NRVERVVN

                          .........210.....
(PRED) CiLV-C_CRD   RASRVSTEGT VNFG-
(PRED) CiLV-C_SJP   RASRVSTEGT INFG-
(PRED) PfGSV_Snp    RVG-----GQ QQFQG
(PRED) PfGSV_BJL    RVG-----GQ QQFQG
(PRED) PfGSV_BSB    RVG-----GQ QQFQG
(PRED) CiLV-C2_Co   RVG-----GQ QSFSG
(PRED) CiLV-C2_Hw   RVG-----GQ QPYTG
(PRED) CiLV-C2_Fla  RVG-----GQ QPYTG
```

Supplementary Figure 3. **Alignment of P24 proteins from cileviruses**. PRALINE software results indicate the **(A)** residue conservation scoring, **(B)** 3-state (H: Helix, E: Strand, C: Coil) secondary structure, **(C)** type of residues, and **(D)** transmembrane structure.
