# Supplementary material for: Passion Fruit Green Spot Virus Genome Harbors a New Orphan ORF and Highlights the Flexibility of the 5′-End of the RNA2 Segment Across Cileviruses
Source: Front Microbiol. 2020 Feb 14;11:206. doi: 10.3389/fmicb.2020.00206 (PMC7033587; doi:10.3389/fmicb.2020.00206)
Supplement: Supplementary file 4 [file Image_4.pdf]

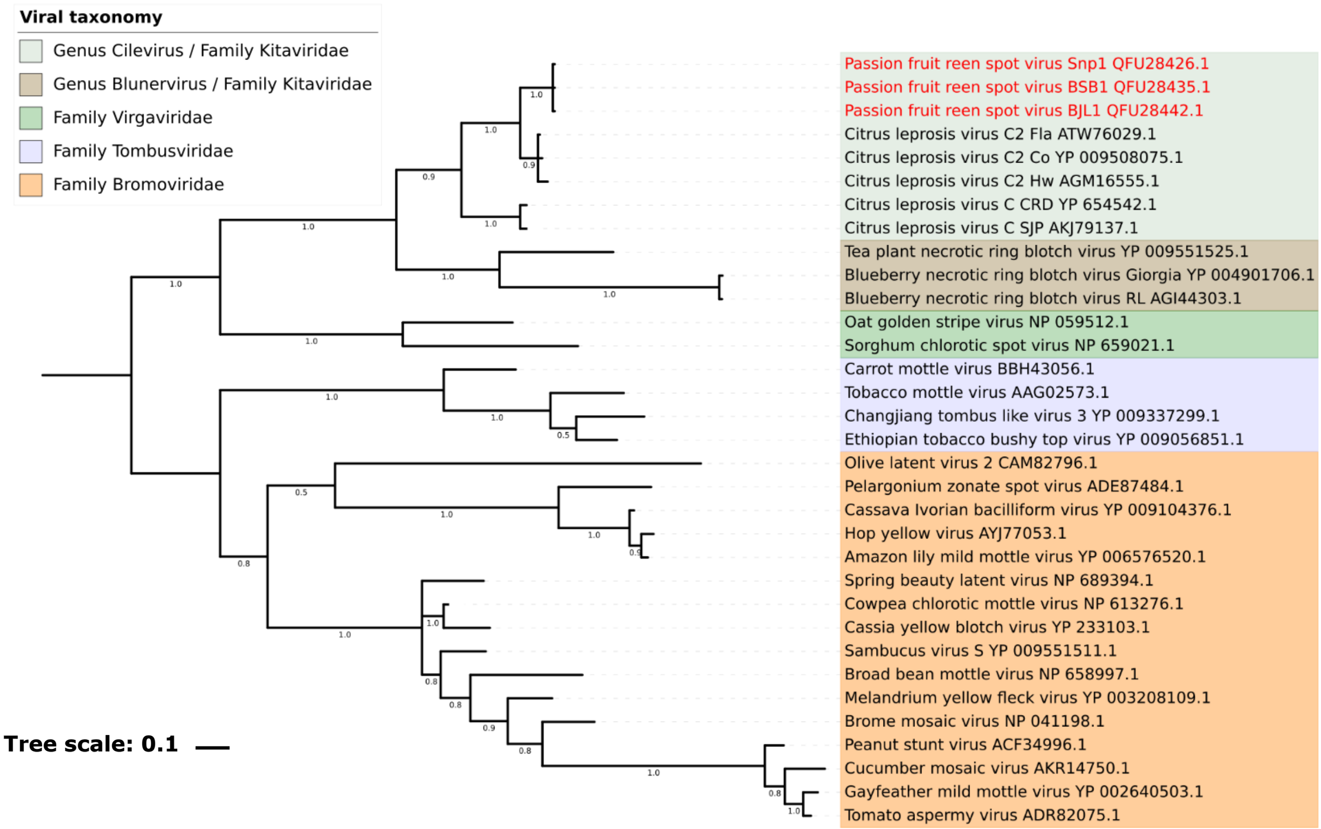

Supplementary Figure S4. Molecular phylogenetic analysis of plant-infecting viruses of the family *Kitaviridae*. The midpoint-rooted Bayesian maximum clade credibility tree was inferred using a Markov Chain Monte Carlo (MCMC) of 6 million generations using the amino acid sequences of movement proteins (MP) of members of the family *Kitaviridae* (genera *Cilevirus* and *Blunervirus*), and other plant infecting viruses of the families *Virgaviridae*, *Bromoviridae,* and *Tombusviridae*. Dataset included 120 positions and its evolutionary history was inferred based on the model LG +G + I +F (Le and Gascuel, 2008). Figures next to nodes indicate values of posterior probability branch support. Scale bar indicates the average number of amino acid substitutions per site. Passion fruit green spot virus sequences are highlighted in red.
