# Supplementary material for: Passion Fruit Green Spot Virus Genome Harbors a New Orphan ORF and Highlights the Flexibility of the 5′-End of the RNA2 Segment Across Cileviruses
Source: Front Microbiol. 2020 Feb 14;11:206. doi: 10.3389/fmicb.2020.00206 (PMC7033587; doi:10.3389/fmicb.2020.00206)
Supplement: Supplementary file 5 [file Table_1.docx]

**Supplementary Table 1**. Set of primers for the secondary sequencing and RACE (Rapid Amplification of cDNA Ends) of the genome of passion fruit green spot virus (PfGSV).

| Name | Primer sequence (5′－3′) | Direction | Target region^a^ | Amplicon size (bp)^a^ |
| --- | --- | --- | --- | --- |
| RNA 1 | | | | |
| 1F | GTGTTATAAATGTACCTGA | F | 520-538 | 909 |
| 1R | CGTATCCAGTATTATATTC | R | 1410-1428 |  |
| 2F | GATTGACGTTTAACATAC | F | 1351-1368 | 752 |
| 2R | GTGACCTATAGTAGTCTCAT | R | 2083-2102 |  |
| 3F | GTAGTTCATGATTCTTCA | F | 2001-2018 | 1377 |
| 3R | CTCGCTTAGTACATTAAC | R | 3360-3377 |  |
| 4F | GTAACTCCTATGAGTACTGT | F | 3231-3250 | 975 |
| 4R | GGTATTGTGTATCTTAACTT | R | 4186-4205 |  |
| 5F | CTTTAACGATAAGTCTGA | F | 4058-4075 | 978 |
| 5R | GAGGATTCAGTTATTAAAC | R | 5017-5035 |  |
| 6F | CTAAGTTTAGCTTATTCCT | F | 4908-4926 | 982 |
| 6R | AAAGTATTGGTAAGGTATC | R | 5871-5889 |  |
| 7F | TCTTATAGGTCTATTCCAC | F | 5775-5793 | 905 |
| 7R | ATCAATGAGTATTTCTCTAC | R | 6660-6679 |  |
| 8F | ATCTGTAGTAAACGAAGTAG | F | 6611-6630 | 973 |
| 8R | TAAACATCTGATGACTTC | R | 7566-7583 |  |
| LigF | AATCTAGCTCAAAACCCACA | F | 6685-6704 | 1869 |
| 10R | AAAGAATCCATAGAAAAC | R | 8536-8553 |  |
| RNA 2 | | | | |
| 1Fa | TATATTGTAAAATGTCTGG | F | 98-116 | 1127 |
| 1Rc | TAACATCAAACTATTTACAC | R | 1205-1224 |  |
| 1Fc | CCATCAATCATTTTATTA | F | 861-878 | 1470 |
| 2R | CTACCAAGATTAAATACG | R | 2313-2330 |  |
| 2F | GCTAGGTTTACTTACAGTC | F | 1377-1395 | 1593 |
| 3R | TAGAAAGTCGAGCTAATA | R | 2952-2969 |  |
| 4F | GATACTTTTGTGTTTATTG | F | 2835-2853 | 545 |
| 4R | TATCCTGAATTGAATATG | R | 3362-3379 |  |
| C6F | CGATATTTGATCAATCCGTT | F | 3310-3329 | 1331 |
| 5R | CTGATAGTATCAACAAAACAC | R | 4620-4640 |  |
| C8F | TTCATCGCAAGTTCGTATACCT | F | 3791-3812 | 910 |
| 5Rb | TTAAACCCCTTCCAGCTG | R | 4683-4700 |  |
| RNA1 5’ RACE | | | | |
|  | CACGCGTGAACGCCTGTCAGTTCTTC | R | 765-790 |  |
|  |  |  |  |  |
| RNA2 5’ RACE | | | | |
|  | GTCCGCTTAATACCATTACGGTCCGAAGAG | R | 583-612 |  |

^a^Data according to the genome of PfGSV isolate Snp1
