# Supplementary material for: Passion Fruit Green Spot Virus Genome Harbors a New Orphan ORF and Highlights the Flexibility of the 5′-End of the RNA2 Segment Across Cileviruses
Source: Front Microbiol. 2020 Feb 14;11:206. doi: 10.3389/fmicb.2020.00206 (PMC7033587; doi:10.3389/fmicb.2020.00206)
Supplement: Supplementary file 6 [file Table_2.docx]

**Supplementary Table 2**. High-throughput sequencing libraries. Composition, viral genome coverage, and largest generated contigs of passion fruit green spot virus genomes obtained from infected passion fruit (*Passiflora* spp.) plants.

| Viral isolate | **PfGSV_Snp1** | | **PfGSV_BSB1** | | **PfGSV_BJL1** | |
| --- | --- | --- | --- | --- | --- | --- |
| Plant  source | *Passiflora* sp. | | | | | |
| Total of reads | 28,998,830 | | 43,249,310 | | 34,260,276 | |
| Genome molecule | RNA1 | RNA2 | RNA1 | RNA2 | RNA1 | RNA2 |
| Lenght (nt) | 8,753 | 4,793 | 8,710 | 4,752 | 8,739 | 4,769 |
| GenBank accession number | MK804171 | MK804172 | MK804173 | MK804174 | MK804175 | MK804176 |
| Viral RNA  matched reads | 312,325 | 510,566 | 296,008 | 1,396,167 | 332,887 | 1,121,130 |
| Percentage of viral specific reads | 1.0 | 1.7 | 0.68 | 3.2 | 0.9 | 3.2 |
|  | 2.8 | | 3.9 | | 4.2 | |
| Mean coverage of viral bases | 9,367 x | 22,052x | 14,842x | 86,525x | 15,817x | 106,838x |
| Length of the largest contig generated by Trinity assembly  (% of the viral genome) | 8,681  (99.1) | 4,767  (99.4) | 8,574  (98.4) | 4,742  (99.7) | 8,715  (99.7) | 4,769  (100) |
